# CARGO: effective format-free compressed storage of genomic information

**DOI:** 10.1093/nar/gkw318

**Published:** 2016-04-29

**Authors:** Łukasz Roguski, Paolo Ribeca

**Affiliations:** 1 Algorithm Development, Centro Nacional de Análisis Genómico, Carrer Baldiri i Reixac 4, Barcelona 08028, Spain; 2 Experimental and Health Sciences, Universitat Pompeu Fabra, Carrer Doctor Aiguader 88, Barcelona 08003, Spain; 3 Integrative Biology, The Pirbright Institute, Ash Road, Pirbright, Woking, GU24 0NF, United Kingdom

## Abstract

The recent super-exponential growth in the amount of sequencing data generated worldwide has put techniques for compressed storage into the focus. Most available solutions, however, are strictly tied to specific bioinformatics formats, sometimes inheriting from them suboptimal design choices; this hinders flexible and effective data sharing. Here, we present CARGO (Compressed ARchiving for GenOmics), a high-level framework to automatically generate software systems optimized for the compressed storage of arbitrary types of large genomic data collections. Straightforward applications of our approach to FASTQ and SAM archives require a few lines of code, produce solutions that match and sometimes outperform specialized format-tailored compressors and scale well to multi-TB datasets. All CARGO software components can be freely downloaded for academic and non-commercial use from http://bio-cargo.sourceforge.net.

## INTRODUCTION

A few typical strategies have been employed so far to implement compressed storage for genomic data:

- Compression tied to some specific datafile format such as FASTQ or SAM (1–5).
- Compression implemented on the top of a pre-existing database engine or data framework as in HDF5, MonetDB or ADAM (6–8).

Usually solutions belonging to the first category are relatively straightforward to implement and optimize, and sufficient for archival purposes. However, they are unsuitable for applications requiring more sophisticated or volatile derived data models, like ‘on-the-fly’ intermediate schemes produced by data analysis workflows that do not necessarily correspond to an available long-term storage format. Solutions in the second category automatically inherit many pros of the framework they build upon, mainly high-level features and seamless integration with other tools. However, they also constrain the user within the boundaries of a possibly cumbersome predefined architectural computational ecosystem, and often turn out to be suboptimal in terms of either storage usage or CPU time consumption.

**Figure 1.**
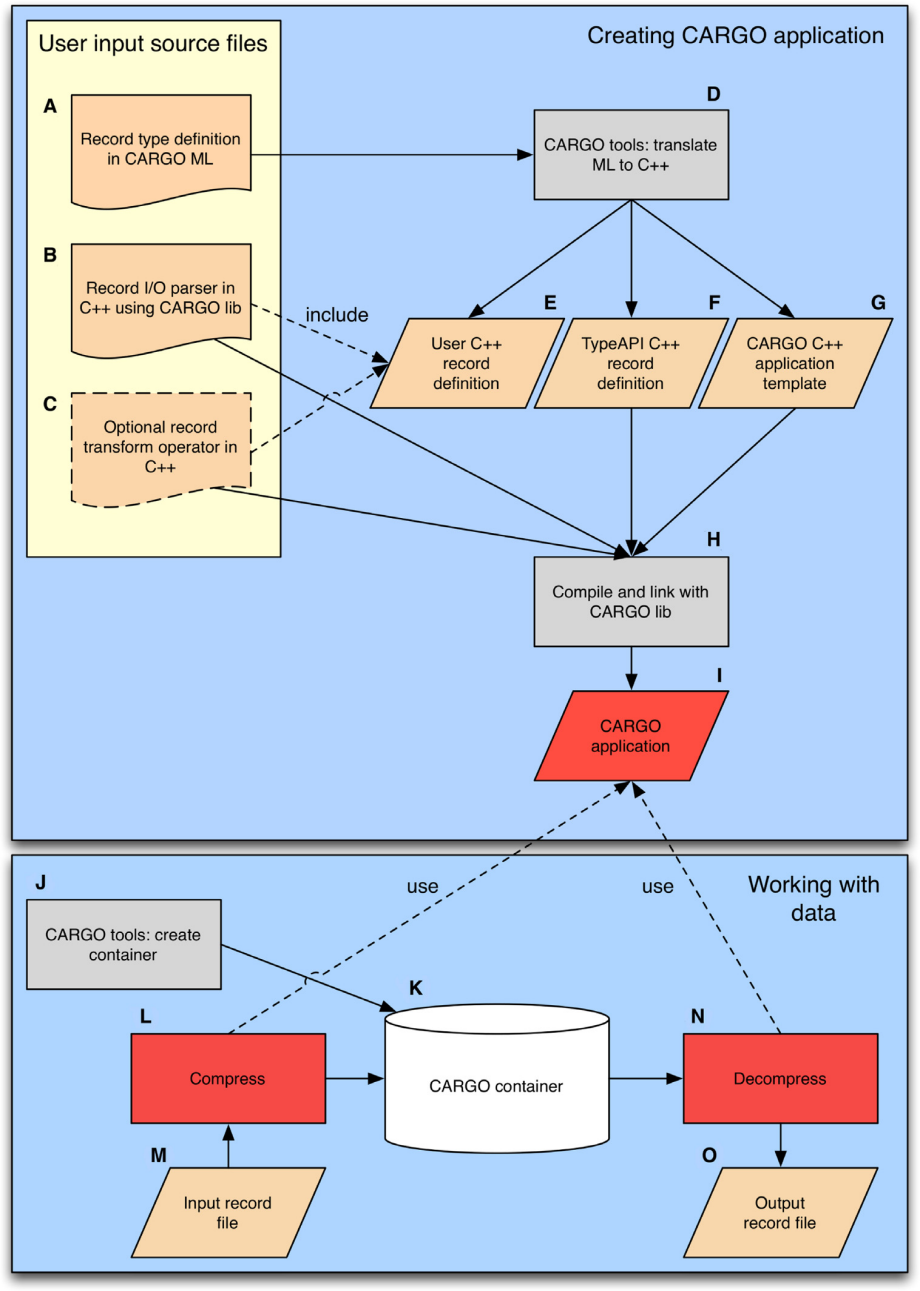
Storing big genomic data in compressed form within the CARGO framework. CARGO is a compressor compiler: all the user has to do in order to produce a family of compressors/decompressors is define a record data type, and specify how to parse/unparse it (see Section INTRODUCTION for a detailed explanation).

**Figure 2.**
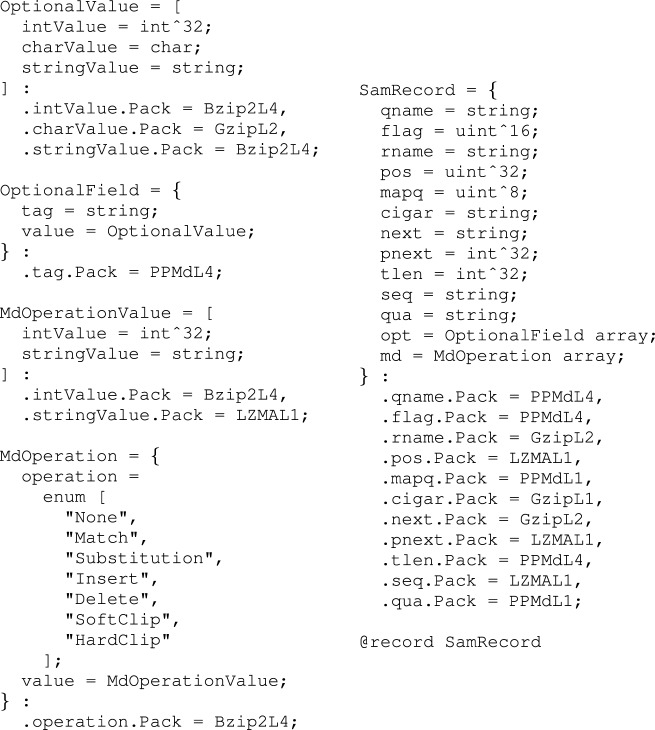
A more realistic SAM record type definition in CARGO meta-language, based on which our best reference-based SAM compressors are implemented. One can notice that different streams are compressed by different methods. This ‘optimal’ configuration was selected based on a number of experiments carried out on different datasets.

Here, we present a different approach, illustrated in Figure 1, that focuses on flexibility and immediate deployability in any local high-performance computing setup. It is based on a few key concepts:

1. We model each dataset as a free-text *header* plus a sequence of structured, and possibly sorted, *records*.
2. As when using fully fledged databases, each record is precisely defined in terms of an expressive domain-specific meta-language (Figure 1A, Section METHODS), i.e. a computer-understandable language that is especially designed in order to describe the problem at hand and has a formal syntax (Supplementary Documentation Section 4). This allows to create rich data types, including arbitrarily nested vectors, records, unions and special data types optimized for the storage of genomic information. From the record specification, in a way which is completely transparent to the user, our framework is able to automatically produce an optimized C++ specification for a compressor/decompressor implementation (Figure 1D–G, Section METHODS). The data fields belonging to the record are analyzed and internally rearranged; fields having the same nature are brought together; and the record is turned into a collection of *streams* which contain homogeneous data and hence can be compressed more effectively, in the spirit of column-oriented databases (Section METHODS). The meta-language allows the user to specify the record in great detail, including the way each field should be compressed. Our framework supports many stream compression methods (at the moment gzip, bzip2, PPMd and LZMA); new methods can be easily added as plug-ins. Parallel multithreaded compression of the streams is transparently provided (Section METHODS).
3. Describing the conceptual ontology of the data in the CARGO meta-language (for instance, a record data type suited to store short-read alignment information as in Figure 2, Section METHODS) is not enough to create a fully working (de)compressor. In order to do so, the user has to provide additional record parsing/unparsing methods written in C++ (see Supplementary Documentation Sections 6 and 8), that specify how a chunk of an input text file (for instance, a line in SAM (1) format) should actually be turned into the abstract record, and vice versa (Figure 1B). Optionally, some parameterized adaptor functions can also be specified, to perform on-the-fly transformations on the record content (for instance, quality downsampling) before compression/after decompression (Figure 1C). Once all the needed inputs for a given format have been provided, they can be compiled by using the standard CARGO toolchain commands (Supplementary Documentation Sections 2, 4 and 8) to produce a compressor/decompressor program in binary form (Figure 1D, H and I).
  Support for several widely used formats (mainly FASTQ and SAM) is provided in the standard CARGO distribution as either precompiled binaries ready to be used or, optionally, suitable input source files as in Figure 1A–C that can be compiled by the user. In other words, optimized CARGO-based compressors for FASTQ and SAM are available out of the box, without any need for the user to develop code or learn the inner workings of CARGO.
  The procedure described at step 3 so far will produce a stand-alone CARGO application—either a compressor able to read some user-specified format and output compressed data to a CARGO container, or a decompressor able to read compressed data from a CARGO container and turn it into some user-specified format. However, a more general alternative is possible, albeit at the price of significantly greater complexity and effort from the user: one can link into one's custom C/C++ code the C++ compression/decompression machinery generated so far, by including both the CARGO library and the format-specific files produced during the previous steps 1 and 2. This allows the development of arbitrarily complex applications. For instance, with this latter technique one would be able to include external libraries and easily read from/write to relevant bioinformatics file formats for which an efficient parser/unparser implementation is already available.
4. Once one or more (de)compressors are available, the user can allocate and populate a CARGO *container* (see Figure 1J–O). A container is a large disk-allocated file designed for the efficient storage of many compressed CARGO streams (Section METHODS). Different formats and different datasets can be written to, or read from, the same container through any of the (de)compressors produced as per the previous step. Albeit some features are not yet implemented, the CARGO tools provide conceptual support for a range of operations on containers, including querying for contained datasets, expansion, shrinkage, concurrent write/read of independent datasets, back-up of meta-data, recovery in case of corruption and so on.
5. If a dataset is presorted according to a criterion specified by the user before being stored in a container, faster range queries on the key—requiring, if *N* is the number of records in the file and *n* the number of records returned by the query, a time of *O*(log*N*+*n*) rather than *O*(*N*) in order to find and extract all the relevant records—become possible for that dataset. This is a generalization of the sort-by-position capability offered by indexed BAM (1) files.

## METHODS

In this section, we describe in more detail how the major features provided by CARGO are implemented.

### How CARGO meta-language record definitions are turned into streams

**Figure 3.**
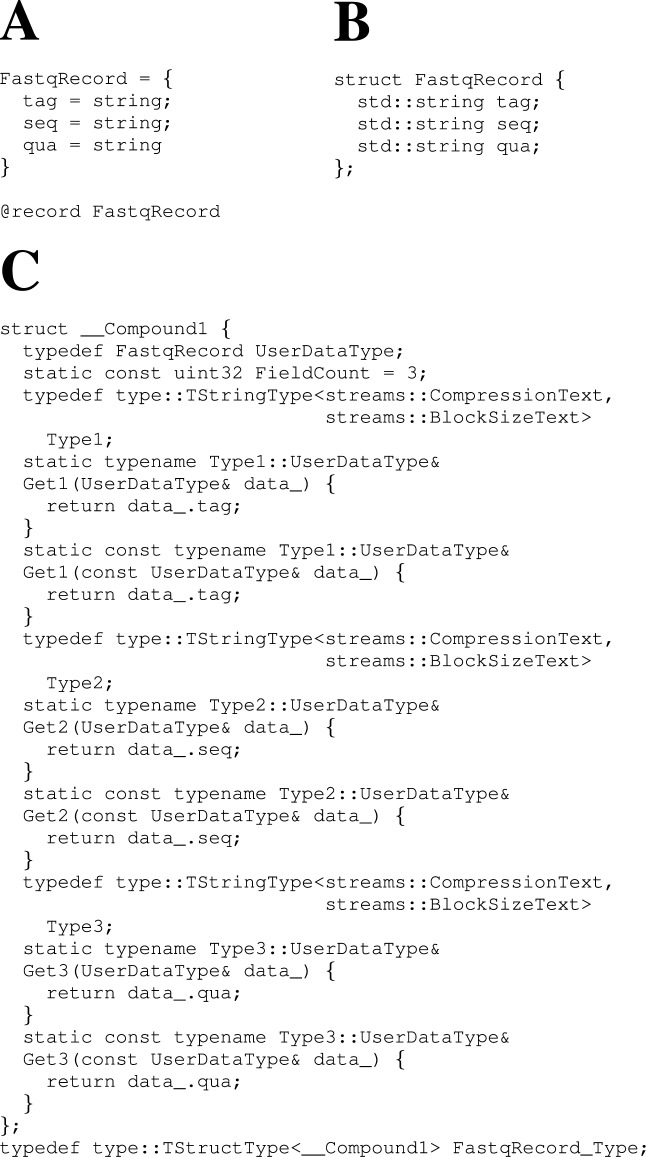
How a type definition in CARGO meta-language is translated into low-level C++ code. **Panel A**: A simple yet complete CARGO meta-language specification for a FASTQ record (as in Figure 1A). **Panel B**: The corresponding C++ record definition automatically generated (as in Figure 1E) by processing the meta-language definition with the CARGO tools (as in Figure 1D). **Panel C**: The C++ TypeAPI definition automatically generated (as in Figure 1F) by the CARGO tools from the meta-language definition. In most cases one does not need to understand, nor use, the automatically generated C++ TypeAPI definition in Panel C in order to be able to create compressors with CARGO.

In order to illustrate how CARGO meta-language is translated to C++ code, we will use a simple proof-of-concept FASTQ record definition (see Figure 3A). More realistic worked-out examples for both FASTQ and SAM format, together with the full definition of the CARGO meta-language in Backus-Naur form, can be found in Supplementary Documentation Section 4.

According to the workflow depicted in Figure 1, the CARGO meta-language record definition (as in Figure 3A) will need to be processed with the CARGO tools. This operation will generate several files containing C++ code. The most relevant ones are:

- A C++ record type definition (see Figures 1E and 3B). It represents the handle by which the user can implement operations on the record such as parsing, transformations and key generation (for sorting and querying).
- A C++ TypeAPI-based record description (see Figures 1F and 3C). The TypeAPI is a core component of the CARGO framework, made of a large set of C++ template classes (see Supplementary Documentation for a full description). They encapsulate the stream I/O management logic by interfacing the C++ type definition with the container functionality. Hence the TypeAPI provides a middle layer between the high-level meta-language description and the low-level data stream representation.
- Some C++ classes providing the skeleton of a record parser and, optionally, functionality to transform records and generate keys to store the data in sorted order (as in Figure 1C). In order to be able to produce a fully working compressor/decompressor, a few missing code pieces need to be manually filled out by the user.

All the C++ files mentioned above are automatically deduced from the provided CARGO meta-language type definition. In particular, both the C++ record type definition and the C++ TypeAPI-based record description can be emitted without any user intervention. This is possible thanks to the CARGO translator application (see Supplementary Documentation for a full description of the program and its options): it parses programs in CARGO meta-language, builds an internal representation of each type in the form of a set of annotated *abstract syntax trees* (ASTs; one per each @record directive present in the meta-code, as in Figure 3A) and turns each AST into valid C++ code. Such code relies upon the TypeAPI classes in order to interface with the CARGO library and so provide access functionality to compressed containers. However, one should note that the TypeAPI is designed to provide an implementation layer automatically invoked by the CARGO meta-language translator, not something whose details should be directly exposed to the user. Most users will never need to look into the TypeAPI-based record description, unless they need to perform some very low-level optimization. For instance, none of the CARGO compressors described in this article required any modification to the machine-generated TypeAPI code.

Even a very simple CARGO meta-code like that of Figure 3A is able to produce optimized compressors/decompressors that rival in performance with the current state of the art (see Supplementary Data Section 1.3). However, more complex file formats and more sophisticated data transformation strategies (like for instance those relying upon reference-based sequence compression) are likely to require more fine-tuned CARGO meta-language record definitions. A fully fledged record type definition, suitable for implementing the high-performance SAM compressor presented in Figure 5 methods 1, 2 and 7, is shown in Figure 2.

### Internal organization of containers

**Figure 4.**
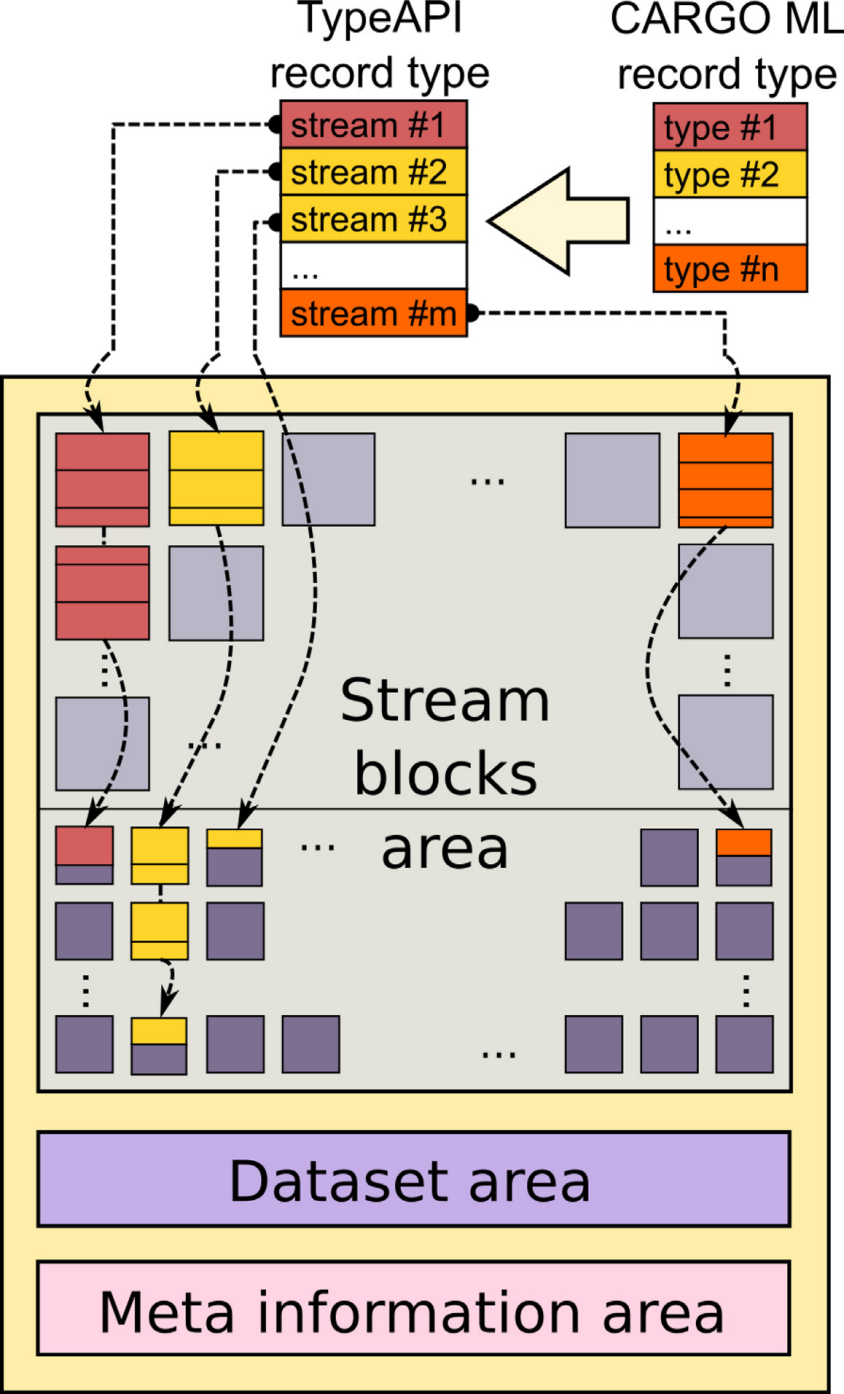
A conceptual representation of low-level information storage in CARGO. Once a meta-language record type has been turned into a C++ TypeAPI definition, the input data are automatically split into a collection of streams. Stream configuration is recorded in the *dataset area*. Streams are stored as linked lists of *blocks*. Blocks can be big or small, small blocks usually being used to store the terminal part of the stream in order to optimize space usage. Block state at any timepoint is stored in the *meta-information area*.

As illustrated in Figure 4, containers are made of three different storage areas:

1. A *stream block area*, where the content of streams is stored. Each stream is conceptually a linked list of separated blocks. Blocks can be either big or small; the sizes of both small and big blocks for each container are configured once and for all when the container is created. Big blocks are used by default in order to minimize disk accesses and improve throughput, whereas small blocks are designed to deal with the last portions of the streams in order to minimize the amount of unused space in big blocks. Additionally, the streams store into blocks checksum information for the contained data at regular intervals.
2. A *meta-information area*. It contains the settings used to generate the container (like block sizes and counts), and a *block allocation table* with information about the occupancy state of each block (which can be either *free*, or *reserved* i.e. currently being written to but not yet finalized, or *occupied*).
3. A *dataset area*. It contains a description of all datasets stored in the container: their type definition and their composition in terms of streams; for each stream, the compression method and a list of block IDs, the number of blocks and all the relevant metrics.

In order to provide for easier backup and recovery, in our current implementation each storage area corresponds to a separate file.

A key design feature of streams is that the blocks composing them do not need to be consecutive. As a result, many different interleaved streams can coexist in the same container, and, provided that a suitable locking mechanism is put in place, many processes can read and write to the same container concurrently.

Overall our container design is very flexible, in that it allows for an easy implementation of many high-level features. For instance, when the amount of needed space is not known in advance a container can be generated with an arbitrary size; however, once all the datasets of interest are stored in it, the container can be shrunk in order to remove unused blocks and facilitate data archival/exchange (see Supplementary Data Section 3.2.2). Although not yet currently implemented, other possible features would be block encryption and resilience to error. The latter would work in a way similar to what is offered by filesystem recovery programs: it would rely on the presence of checksum information within the blocks in order to detect possible corruptions due to hardware errors occurring in the physical layer of the storage, and would allow the reconstruction of the whole dataset except for the records directly hinging on the affected block.

As each stream is saved as an independent list of blocks, after a record had been parsed and optionally transformed using a user-specified adaptor function, each block can be compressed independently (the same holds in the reverse order for decompression). Considering that record parsing and transformation are usually not the data-processing bottlenecks, this scheme offers big room for implementing transparent parallelization within the CARGO library. In our current implementation, we follow a standard producer/consumer paradigm and dispatch a small slice of the input records to each processing thread. After streams have been parallelized in this way, I/O throughput usually becomes the performance bottleneck. This is why selecting big block sizes can improve performance, especially on the network-distributed filesystems usually found in high-performance computing environments.

In addition to the custom record transformation functionality, another important feature offered by CARGO is the possibility of specifying an arbitrary custom sorting function defined in terms of the record, and then performing range queries on such a generated key. In this case streams will contain additional information stored in the dataset area, consisting of keys sampled at regular intervals. This allows us to implement locating searches by simple bisection methods. Once blocks within the query range have been identified, subsequent data extraction can be easily parallelized.

## RESULTS

In Supplementary Documentation Section 8 we demonstrate in a step-by-step tutorial how families of compressors/decompressors from/to formats widely used in the bioinformatics of high-throughput sequencing, such as FASTQ (9) and SAM, can be implemented with CARGO. In fact, several implementations of increasing complexity and sophistication are included in the standard CARGO distribution. Our full results are presented in Supplementary Data Section 1; here we focus on two benchmarks testing recompression of SAM files, although similar results hold true for FASTQ files. In general our framework provides unprecedented flexibility, excellent performance and good scalability.

### Recompression of small SAM files

**Figure 5.**
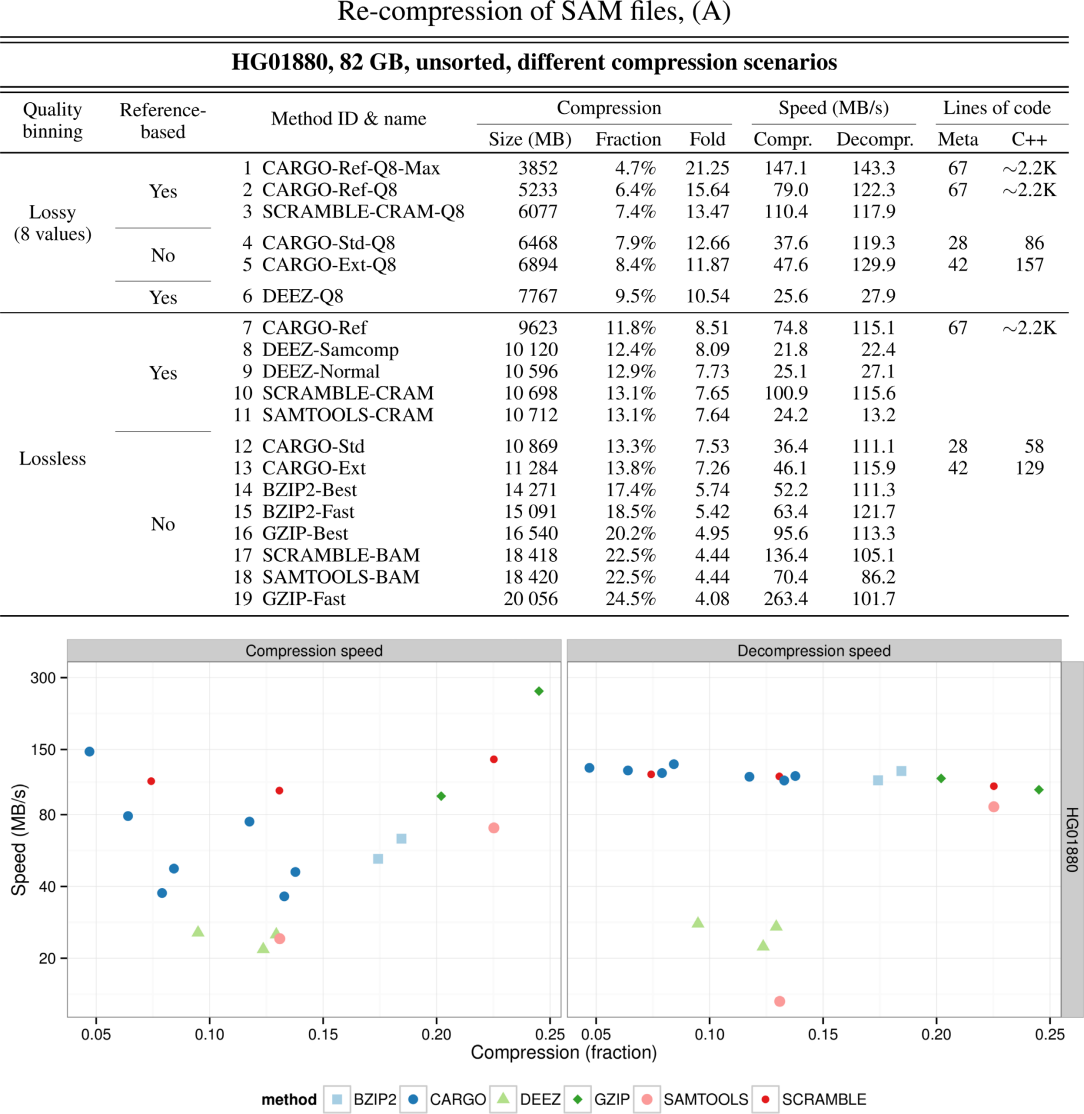
Several methods for recompressing SAM files benchmarked on a 82 GB file containing DNA-sequencing data. The value of the two main technical parameters for each method—i.e. whether qualities have been downsampled to eight possible values following the Illumina binning scheme, and whether the method uses reference-based compression for DNA sequences—are shown in the first and second column of the table, respectively. In the names of lossy methods, ‘Max’ means that read names and optional SAM fields have been discarded as well. The precise definition of each method can be found in Supplementary Data appendix B. ‘Fraction’ is the ratio between the sizes of compressed and original file; ‘Fold’ is the ratio between the sizes of original and compressed file. For each CARGO method, the number of source code lines needed to implement it on the top of the CARGO library is also shown.

Figure 5 illustrates our results when recompressing a 82 GB SAM file with different methods (dataset HG01880 from the 1000 Genome Project (10), see Supplementary Data for a complete description of all methods).

Perhaps the most striking feature of our approach is that in general very little code is required to achieve results that are comparable to, or better than, what state-of-the art compressors can obtain. In fact about 30 lines of CARGO meta-code supplemented with less than 90 lines of C++ code on the top of our framework are sufficient to implement a SAM compressor achieving compression levels similar to those offered by the recently published DeeZ (5) and being several times faster at both compression and decompression (Figure 5, methods 4 and 12 versus methods 6, 8 and 9).

With some more code (70 lines of CARGO meta-code and about 2000 lines of C++ code) one can implement a fully-fledged SAM compressor offering advanced features like reference-based sequence compression and optional lossy Illumina 8-level quality rebinning; both compression levels and (de)compression speed are on par with those provided by a recent version of HTSLib-based (11) CRAM (12) implementation (Figure 5, method 2 versus 3 and method 7 versus 10). In addition, by changing a few lines in the meta-code description we can easily generate a family of compressors that are suitable for different scenarios (like: slower compression but faster decompression; or both slower compression and decompression but smaller final size).

Our best lossy quality resampling scheme uses the same compression setups as CRAM, additionally stripping out read names and optional SAM fields as in the initial implementation of CRAM; it produces archives that are about two times smaller than the corresponding CRAM ones, and is able to compress/decompress faster than CRAM (Figure 5, method 1 versus 3).

Of note, although performing the same function our family of compressors and those developed so far to operate on fixed-format files differ in a number of fundamental philosophical points. In our case, rather than from highly optimized *ad hoc* code, good performance stems as a straightforward by-product from simple design choices, in particular:

- Automatic data compartimentalization into separate streams offered by the framework (Section METHODS).
- The fact that we use an optimized set of methods in order to compress SAM streams, as described in Figure 2. Such set of methods was determined experimentally by running on a training dataset many different CARGO compressors—each one generated by simply specifying different compression methods for the streams in the last ten lines or so of the meta-code specification of Figure 2—and selecting the solution offering the best trade-off between speed and compression rate. Actually, the fact that we were able to achieve this optimization by just changing a few lines of high-level code neatly demonstrates one of the most interesting features of CARGO, i.e. the possibility of quickly prototyping different compression configurations without any need for redesigning the whole compressor or having to bother about low-level optimizations.
- In the most sophisticated of our methods, some data transformations that we are applying to the input data (see the source code for methods 1, 2 and 7 of Figure 5 in the standard CARGO distribution for a precise definition).
- The fact that we are able to explicitly tune the size of input data buffers, thus achieving a better overall compression ratio.
- Automatic user-transparent multithreading offered by the framework (see Section METHODS).

In addition while, say, a SAM file compressed to CRAM needs a specific tool for the semantics of its content to be successfully recovered, the same data compressed within our framework does not. For instance, we fully parse the SAM record including its optional fields, and every component of the data structure is subsequently available to (de)compressing programs on the C++ side of the CARGO implementation. It would be perfectly possible to output a different format (for instance, one including less or additional fields) starting from the same database.

### Recompression of large SAM files

**Figure 6.**
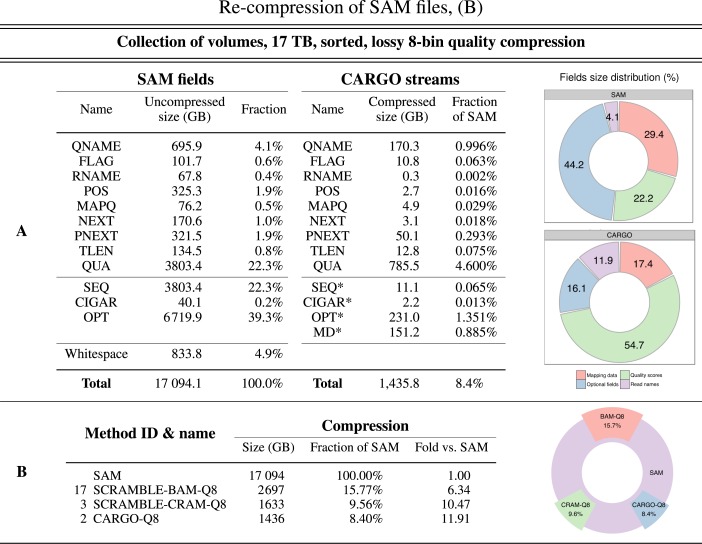
Three methods for recompressing SAM files benchmarked on a collection of files containing 17 094 GB of DNA-sequencing data. ‘Fraction’ is the ratio between the sizes of compressed and original file; ‘Fold’ is the ratio between the sizes of original and compressed file. **Panel A**: Breakdown of SAM file size by SAM field (see (1) for the precise definition) and of CARGO container size by CARGO streams (see Supplementary Data for a precise definition—when a CARGO stream does not exactly correspond to a SAM field, it is marked with an asterisk). **Panel B**: Benchmark results at a glance.

In addition, our approach can easily scale up to multi-TB datasets. Figure 6 illustrates the results of an experiment whereby we compress a large collection of SAM volumes from the 1000 Genome Project (17 TB uncompressed total, see Supplementary Data Section 2 for the complete list of archives) into:

1. A single CARGO container using our second most effective compression scheme (same as method 2 of Figure 5).
2. A single BAM archive recompressed with SCRAMBLE (same as method 17 of Figure 5 with additional quality score binning activated, see Supplementary Data for the precise parameters used).
3. A single CRAM archive recompressed with SCRAMBLE (same as method 3 of Figure 5).

The CARGO scheme was selected as a reference because it is directly comparable with BAM or CRAM, provided that suitable parameters are selected for those methods (see Supplementary Data for a full list). CARGO streams were stored sorted by genomic position, in order to make the archive searchable and mimic the search-by-position capabilities offered by BAM/CRAM formats.

In line with the results presented in the previous section for smaller files, also in this case our best method achieved a compression rate that is almost twice as much as that of BAM's, and significantly better than the one provided by CRAM. Notwithstanding, and depending on the size of the range queried, the time needed to query a CARGO container is either slightly worse, or comparable to, the one needed to query the much less compressed BAM container; and it is much better than the querying time for a CRAM container (see Supplementary Data Section 1.2). Finally, the data throughput obtained by CARGO is several times higher than what SAMtools implementations of either BAM or CRAM format can offer at the moment (see Supplementary Data Sections 1.1 and 1.2), making CARGO an ideal storage tool for high-performance downstream applications.

## DISCUSSION

In general, what our approach can achieve goes far beyond the compression of formats like FASTQ or SAM, offering many advantages with respect to most solutions currently available in the field of genomics:

1. In the spirit of database format design and different from rigid, complicated, ambiguously defined file-based formats such as SAM, our data representation is based on an *abstract description of the content* defined in terms of a specialized domain-specific language. This results in flexibility, in fast reaction to new genomic technologies or specific data analysis frameworks requiring amendments to the data structure (whereas solutions like SAM would require a redefinition of the format and its re-implementation in all downstream tools), in meaningful data interchange based on the semantics of the record description, in efficient automatic optimization and multithreading of CARGO applications.
2. Our framework is high-level and modular, thus fostering *parsimonious implementations* that require very little amounts of CARGO meta-language and C++ code to produce optimized compressed storage systems for complex data structures: the user does not need to install, or delve into the complicated technical details of, a general-purpose database system/data framework.
3. Our simple and concise scheme allows for a *versatile approach to storage*: in order to identify the combination that best matches the requirements of an application, the user can easily try out different data structures and combinations of compression methods for each data field; moreover, the stock CARGO engine can be easily extended by incorporating new compression methods and/or support for accelerated hardware where available. For instance—although we did not pursue such an approach in this paper—in addition to the traditional generalist methods (like gzip, bzip2, PPMd and LZMA) that we offer so far in order to compress streams, we might provide more specialized routines for the compression of DNA sequence (like the one in (13)) or sequencing qualities (see for instance (14)) that have been developed in the recent past. In principle, by combining such building blocks and more sophisticated transformations applied to the data streams one might be able to replicate in CARGO even complex and very optimized file-specific compression methods. Such a strategy might be able to produce even better results than what we presented in this paper, while at the same time retaining all the high-level features offered to the user by the CARGO infrastructure.
4. With its *container-based approach* the system offers a simple way to store analysis intermediates without cluttering the storage with too many files, which is usually very expensive on PB-scale filesystems: this helps improving overall performance on computing clusters. In fact, and provided that enough space is available, the user is able to store transparently in the same container an arbitrary number of CARGO-compressed datasets originating from possibly different file formats. For instance, the raw data generated prior to base calling by a third-generation sequencing technology like Pacific Bioscience or Oxford Nanopore, the sequences derived from such data, the alignments for the sequences and further data of any kind resulting from the subsequent stages of the analysis pipeline could all straightforwardly coexist in the same container. Together with the property described at point 1 above, i.e. the fact that in CARGO one can easily define new formats able to represent nonstandard volatile data structures generated during the intermediate stages of data analysis, it is easy to see that CARGO might be a potentially very interesting candidate whenever one needs a comprehensive compressed storage solution for complex analysis workflows.

As our results stem from a few lines of application-specific code, they are just a glimpse of what CARGO can offer to the field of high-throughput genomics. Bioinformatics has been traditionally plagued by inflexible file formats that constrain effective data exchange. Hopefully our work will demonstrate to the community that using a different, more flexible and high-level approach does not necessarily imply having to forgo the potential benefits—like suitability for high-performance computation and optimization—offered by more traditional solutions.

## Supplementary Material

SUPPLEMENTARY DATA
