# Supplementary material for: CARGO: effective format-free compressed storage of genomic information
Source: Nucleic Acids Res. 2016 Apr 29;44(12):e114. doi: 10.1093/nar/gkw318 (PMC4937321; doi:10.1093/nar/gkw318)
Supplement: SUPPLEMENTARY DATA [file supp_44_12_e114__index.html]

CARGO: effective format-free compressed storage of genomic information — SUPPLEMENTARY DATA 

# CARGO: effective format-free compressed storage of genomic information

## SUPPLEMENTARY DATA

- SUPPLEMENTARY DATA
- SUPPLEMENTARY DATA
