# Supplementary material for: CARGO: effective format-free compressed storage of genomic information
Source: Nucleic Acids Res. 2016 Apr 29;44(12):e114. doi: 10.1093/nar/gkw318 (PMC4937321; doi:10.1093/nar/gkw318)
Supplement: SUPPLEMENTARY DATA [file supp_gkw318_nar-02786-met-n-2015-File012.pdf]

CARGO Supplementary

Release 0.7

Lukasz Roguski, Paolo Ribeca

March 31, 2016

1 Supplementary data **1**
1.1 SAM format compression benchmarks . . . 1
1.2 Queryable large-scale SAM format benchmarks . . . 5
1.3 FASTQ format compression benchmarks . . . 10
1.4 Memory and performance scalability of multithreaded CARGO-based compressors . . . 17
1.5 References . . . 20

2 Appendix A - datasets **21**
2.1 SAM format compression benchmarks . . . 21
2.2 Queryable large-scale SAM format benchmarks . . . 21
2.3 FASTQ format compression benchmarks . . . 25

3 Appendix B - tools invocation **27**
3.1 SAM format compression benchmarks . . . 27
3.2 Queryable large-scale SAM format benchmarks . . . 31
3.3 FASTQ format compression benchmarks . . . 34
3.4 Memory and performance scalability of multithreaded CARGO-based compressors . . . 37

CHAPTER

ONE

SUPPLEMENTARY DATA

1.1 SAM format compression benchmarks

In this section we describe in detail how our SAM format compression ratio and throughput benchmarks were performed. We compare the results obtained by several *CARGO* implementations, each one having a different degree of sophistication and complexity, to those of several state-of-the-art SAM format-specific compressors.

1.1.1 Data set

The test data sets consist of mapped sequences of *H. Sapiens* individuals, which were downloaded from the 1000 Genomes Project in BAM [1] format. We decompressed files and converted them to the SAM format using *SAMtools* version 1.1. Some test scenarios use reference-based compression techniques and thus require the original FASTA reference file which the sequencing reads have been mapped to – in the case of the 1000 Genomes Project the *GRCh37* Human Genome assembly was used, which we also downloaded from the Project's repository (see **Appendix A**).

1.1.2 Reference compressors

The binaries for reference compressors were downloaded from their official websites or compiled from source with default options set in build scripts.

All applications were tested using 8 processing threads (whenever multi-threading is supported by the application).

GZIP

We used version 2.3.1 of *pigz*, a parallel implementation of the *gzip* compression tool (available at http://zlib.net/pigz/). The application was tested in 2 configurations: *GZIP-FAST* providing a good compression ratio together with fast performance, and *GZIP-BEST* providing the highest compression ratio.

BZIP2

We used version 1.1.8 of *pbzip2*, a parallel implementation of the *bzip2* compression tool (available at http://compression.ca/pbzip2/). The application was tested in 2 configurations: *BZIP2-FAST* providing a good compression ratio together with fast performance, and *BZIP2-BEST* providing the highest compression ratio.

SAMTOOLS

We used *SAMtools* version 1.1 (available at http://www.htslib.org/); it implements both BAM and CRAM [2] formats. Similar to the newest versions of *SAMtools*, it is based on *HTSLib*. We tested 2 configurations of the application: *SAMTOOLS-BAM* implementing the standard SAM-to-BAM format compression, and *SAMTOOLS-CRAM* implementing reference-based compression and storing the data in CRAM format.

SCRAMBLE

We used version 1.13.7 of *sCRAMble* [3], a SAM/BAM/CRAM format conversion toolkit (available at http://sourceforge.net/projects/staden/files/io_lib/). We tested 3 configurations of the application: *SCRAMBLE-BAM* implementing the standard SAM-to-BAM format compression, *SCRAMBLE-CRAM* implementing reference-based compression, and *SCRAMBLE-CRAM-Q8* implementing both reference-based compression alongside with the Illumina Q-scores reduction [4] scheme. The first configuration outputs a BAM file, the two latter ones CRAM.

DEEZ

We tested 3 configurations of *DeeZ* [5] (available at http://sfu-compbio.github.io/deez/). *DEEZ-NORMAL* uses default compression parameters, *DEEZ-SAMCOMP* uses a *sam_comp*-compatible [6] compression method and *DEEZ-Q8* uses the default compression method alongside with the Illumina Q-scores reduction scheme.

1.1.3 *CARGO*

We also tested a number of different *CARGO* implementations of the SAM format. The source code for them is available in the standard *CARGO* distribution in the directory `cargo/examples/sam/` and the pre-compiled binaries can be found at `cargo/examples/bin/`. To build and test the executables for all the implementations one needs the following tools: `cargo_translate` (generates C++ files from record definition in *CARGO* meta-language) and `cargo_tool` (allows container management). They are available in the directory `cargo/tools/`.

The examples were all compiled from source and tested using as runtime parameters 8 processing threads and a 64 MB block for the input file buffer.

A more detailed description about how the examples were implemented, how they can be compiled step-by-step and which command-line parameters should be used with them is available in the **Supplementary Documentation**.

Container configuration

Before each test, a temporary container was created, setting the available compressible storage space up to a size of 17.7 GB (for details about the creation of the container see **Appendix B**).

Size measurements

The space occupancy results reported for *CARGO* are those provided by `cargo_tool` about the cumulative size of the compressed dataset. As containers are usually arbitrarily big and can contain more than a single dataset, this might be considered as an indirect measurement of the size of the dataset. However, containers can be shrunk to eliminate free blocks and thus reclaim unused space. In the current implementation, the difference between the actual size of the compressed dataset and the size of the container having been shrunk (which depends on the container block size selected by the user and the amount of block padding inside the container due to data granularity) will usually be negligible with respect to the large size of the compressed dataset, as corroborated by many shrinkage tests we conducted. Being this the case, we finally decided to skip the shrinkage step altogether, and directly report the size of the compressed database instead.

The size of the compressed dataset can be reported either by running the compressor with provided verbosity switch flag or by using `cargo_tool` - the syntax for the command line invocation of all considered tools can be found in **Appendix B**.

CARGO methods

CARGO-SAM-STD

CARGO-SAM-STD is a simple, proof-of-concept SAM format file compressor, where most of the SAM fields are represented either as a string or an integer and compressed using different schemes – the source code for this example is available in the directory `cargo/examples/sam-std/` of the standard *CARGO* distribution, and a pre-compiled binary in `cargo/examples/bin/cargo_samrecord_toolkit-std`. This solution was tested in 2 configurations: lossless *CARGO-SAM-STD*, and lossy *CARGO-SAM-STD-Q8* implementing the Illumina Q-scores reduction scheme.

CARGO-SAM-EXT

CARGO-SAM-EXT is an extended version of the *CARGO-SAM-STD* example, which, in addition to the methods described in *CARGO-SAM-STD*, also performs tokenization of the optional fields instead of compressing them together as one long string. The source code for this example is available in `cargo/examples/sam-ext/`, and the pre-compiled binary can be found at `cargo/examples/bin/cargo_samrecord_toolkit-ext`. This solution was tested in 2 configurations: lossless *CARGO-SAM-EXT*, and lossy *CARGO-SAM-EXT-Q8* implementing the Illumina Q-scores reduction scheme.

CARGO-SAM-REF

CARGO-SAM-REF is a more advanced lossless SAM format compressor, which in addition to the methods used in *CARGO-SAM-EXT* also implements:

- more complex tokenization of the SAM optional fields,
- internal alignment description in terms of a special combination of `SEQ` SAM field, `CIGAR` field and `MD` optional field,
- reference-based sequence compression,
- transformations of several SAM numerical fields including `TLEN` and `PNEXT`.

Additionally, we have two more lossy schemes: *CARGO-SAM-REF-Q8* implements the Illumina Q-scores reduction transformation on the top of the previous lossless manipulations, while *CARGO-SAM-REF-Q8-MAX* also discards both the `QNAME` field and the optional fields `OPT` (keeping intact only the alignment mismatch information defined by the `MD` tag).

The source codes for this example are available in the directory `cargo/examples/sam/sam-ref/` of the *CARGO* distribution, while the pre-compiled binares, one for each different test case, can be found as `cargo/examples/bin/cargo_samrecord_toolkit_`{ `ref`, `ref-q8`, `ref-q8-max` }.

1.1.4 Test setup

The experiments were performed on a server machine equipped with four 8-core AMD OpteronTM 6136 2.4GHz CPUs, 128 GB of RAM and a RAID-5 disk matrix containing 6 non-solid state HDDs.

1.1.5 Results

In this section we present the results of our benchmarks on the compression of SAM format. In the tables below the **Ratio** column is computed as *original SAM size / compressed size*, while **C/size** is the size of the compressed dataset in MB. The **C/speed** and **D/speed** fields represent compression and decompression speed in MB/s computed as the *original SAM size / (de)compression time*, while **C/time** and **D/time** are the total compression/decompression processing times in seconds.

Here it's important to clarify that when testing the *CARGO-REF-Q8-MAX* solution the decompression speed is calculated in a different manner, i.e. *decompressed SAM size / decompression time*, as the resulting file size differs from the original one – the `QNAME` field values and non-alignment related `OPT` field values are discarded.

Additionally, in **Fig. 1.1** we show how our *CARGO*-generated solutions and other SAM format-specific compressors perform in terms of both (de)compression speed and ratio.

Table 1.1: Compressing file HG00306 from the 1000 Genomes Project with several methods. Initial SAM file size: 68.3 GB

Method	Ratio	C/size	C/speed	D/speed	C/time	D/time
CARGO-REF-Q8-MAX	16.99	4017	109	116	626	335
CARGO-REF-Q8	13.13	5199	74	121	921	562
SCRAMBLE-CRAM-Q8	12.17	5608	118	201	579	340
CARGO-STD-Q8	11.26	6061	38	130	1788	527
CARGO-EXT-Q8	10.52	6490	44	126	1547	541
DEEZ-Q8	10.44	6537	24	29	2822	2328
CARGO-REF	7.85	8699	70	132	973	518
DEEZ-SAMCOMP	7.60	8976	22	22	3123	3078
SCRAMBLE-CRAM	7.33	9318	113	180	603	379
DEEZ-NORMAL	7.29	9369	26	28	2649	2467
SAMTOOLS-CRAM	7.29	9364	27	10	2522	6601
CARGO-STD	7.13	9579	37	125	1853	544
CARGO-EXT	6.83	9990	43	123	1600	554
BZIP2-BEST	5.57	12246	58	189	1186	360
BZIP2-FAST	5.23	13062	68	219	1008	311
GZIP-BEST	4.82	14155	93	162	733	421
SAMTOOLS-BAM	4.31	15830	72	86	943	792
SCRAMBLE-BAM	4.31	15829	172	149	397	457
GZIP-FAST	3.96	17243	311	151	219	452

Table 1.2: Compressing file HG01880 from the 1000 Genomes Project with several methods. Initial SAM file size: 81.9 GB

Method	Ratio	C/size	C/speed	D/speed	C/time	D/time
CARGO-REF-Q8-MAX	21.25	3852	147	143	556	364
CARGO-REF-Q8	15.64	5233	79	122	1035	669
SCRAMBLE-CRAM-Q8	13.47	6077	110	118	741	694
CARGO-STD-Q8	12.66	6468	38	119	2178	686
CARGO-EXT-Q8	11.87	6894	48	130	1720	630
DEEZ-Q8	10.54	7767	26	28	3201	2937
CARGO-REF	8.51	9623	75	115	1094	711
DEEZ-SAMCOMP	8.09	10120	22	22	3756	3662
DEEZ-NORMAL	7.73	10596	25	27	3257	3024
SCRAMBLE-CRAM	7.65	10698	101	116	811	708
SAMTOOLS-CRAM	7.64	10712	24	13	3389	6210
CARGO-STD	7.53	10869	36	111	2250	737
CARGO-EXT	7.26	11284	46	116	1776	706
BZIP2-BEST	5.74	14271	52	111	1568	735
BZIP2-FAST	5.42	15091	63	122	1291	672
GZIP-BEST	4.95	16540	96	113	856	722
SCRAMBLE-BAM	4.44	18418	136	105	600	778
SAMTOOLS-BAM	4.44	18420	70	86	1163	949
GZIP-FAST	4.08	20056	263	102	310	805

Table 1.3: Compressing file HG03780 from the 1000 Genomes Project with several methods. Initial SAM file size: 75.5 GB

Method	Ratio	C/size	C/speed	D/speed	C/time	D/time
CARGO-REF-Q8-MAX	16.37	4611	169	125	447	299
CARGO-REF-Q8	13.15	5738	88	140	857	537
SCRAMBLE-CRAM-Q8	11.93	6329	112	117	674	645
CARGO-STD-Q8	10.67	7071	39	125	1948	606
DEEZ-Q8	10.45	7220	27	28	2818	2657
CARGO-EXT-Q8	10.21	7392	47	132	1615	572
CARGO-REF	7.40	10202	78	125	962	605
SCRAMBLE-CRAM	7.22	10451	113	121	670	626
SAMTOOLS-CRAM	7.20	10486	25	12	3046	6083
DEEZ-SAMCOMP	7.00	10786	20	21	3735	3569
DEEZ-NORMAL	6.76	11168	24	26	3121	2866
CARGO-STD	6.53	11550	37	111	2037	678
CARGO-EXT	6.37	11855	44	110	1697	683
BZIP2-BEST	5.10	14799	54	123	1395	614
BZIP2-FAST	4.86	15540	65	111	1169	681
GZIP-BEST	4.57	16499	104	120	725	631
SCRAMBLE-BAM	4.12	18321	139	98	545	767
SAMTOOLS-BAM	4.12	18322	71	85	1061	889
GZIP-FAST	3.84	19676	208	103	362	730

1.2 Queryable large-scale SAM format benchmarks

In this section we describe in detail how our large-scale queryable SAM format benchmarks were performed. We compare the results of a typical *CARGO* implementation with those obtained by the *SAMtools* implementations of

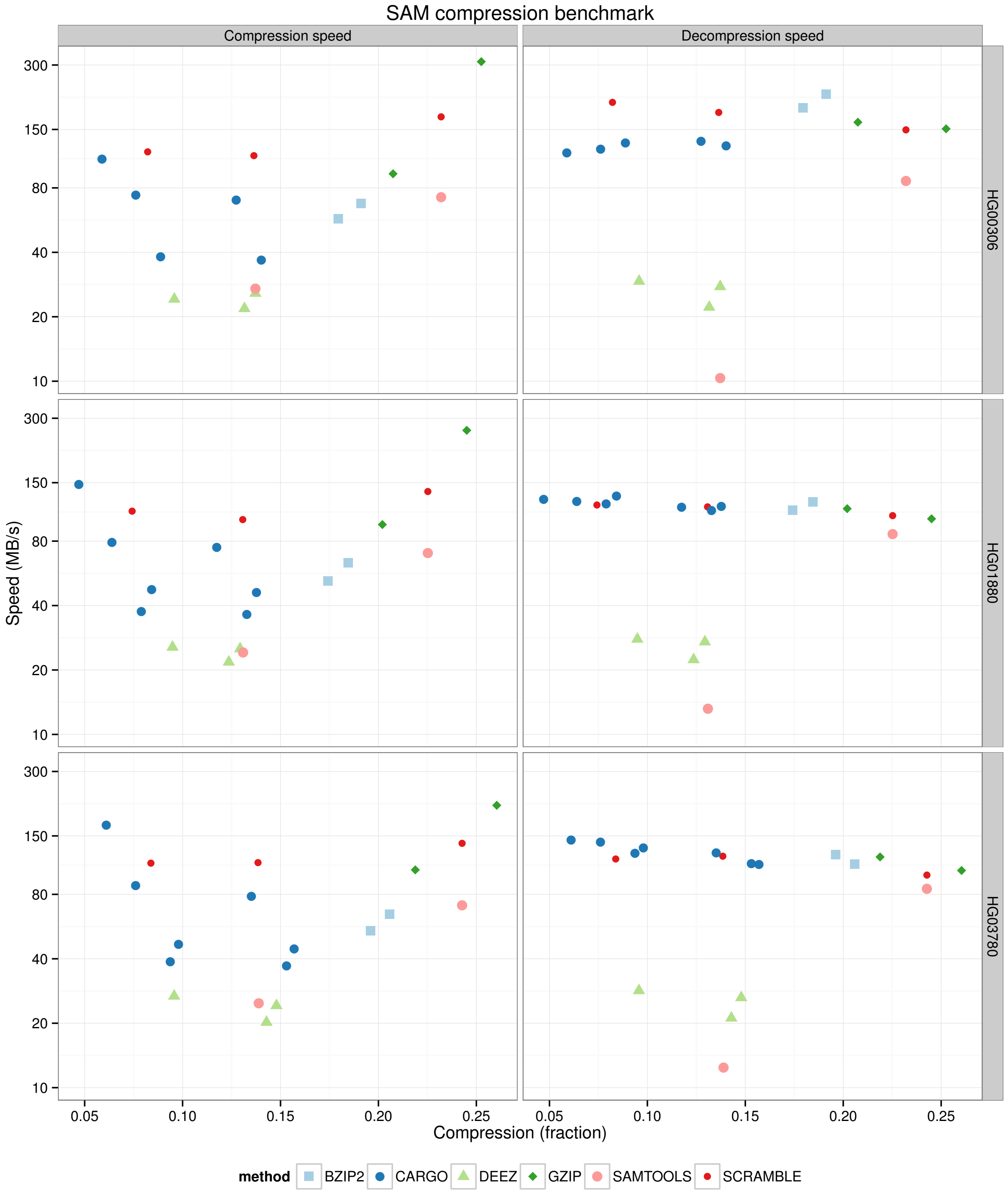

Fig. 1.1: Compression of SAM format: results of throughput vs. ratio benchmarks

both BAM and CRAM formats (the syntax for command line invocation of all considered tools can be found in **Appendix B**). We report on the achieved compression rates, the size of the underlying compressed streams and the range querying times of the compressed datasets.

1.2.1 Data sets

The test data set consist of 2 collections of several BAM files. The first collection (to which we will refer as *small volume* from now on) consists of 8 BAM files for a total compressed size of 209 GB (880 GB of decompressed SAM); the second collection is made of 157 BAM files for a total compressed size of 4.1 TB (17.1 TB of decompressed SAM). All BAM files were publicly available from download on the 1000 Genomes Project repository, and should be regarded as an essentially random selection of the available data (see: **Appendix A** for the complete list of the files in each collection).

1.2.2 Data preparation

First all BAM files in each collection were merged into a single large and sorted BAM volume (labelled as "BAM") using the *SAMtools* toolkit (`merge` subcommand). In the next step, the volumes were re-compressed

- into another BAM archive (labelled as "BAM-Q8") with *sCRAMble*, applying the Illumina Q-scores reduction scheme
- into a CRAM archive (labeled as "CRAM") with *sCRAMble*, applying the Illumina Q-scores reduction scheme
- into a *CARGO* archive, using implementation *CARGO-SAM-REF-Q8*.

In all cases, the records in the archives were stored in sorted order, indexed by chromosome and position.

1.2.3 *CARGO*

For this test we used a *CARGO-SAM-REF-Q8* method – that is, the SAM format compressor implementing reference-based compression and the Illumina Q-scores reduction scheme that was benchmarked in the previous section. The application source code is available in the standard *CARGO* distribution in the directory `cargo/examples/sam/sam-ref`, and the pre-compiled binaries can be found at `cargo/examples/bin/cargo_samrecord_toolkit-ref-q8`.

Container configuration

For each data collection, we created a container with a total available space being 10% of the total uncompressed input data to be stored – respectively up to 1.72 TB (*large volume*) and 86.4 GB (*small volume*). This is enough to accommodate the compressed datasets produced by all *CARGO* methods.

Size measurements

The space occupancy results reported for *CARGO* are those provided by `cargo_tool` about the cumulative size of the compressed dataset. As containers are usually arbitrarily big and can contain more than a single dataset, this might be considered as an indirect measurement of the size of the dataset. However, containers can be shrunk to eliminate free blocks and thus reclaim unused space (see **Appendix B**). In the current implementation, the difference between the actual size of the compressed dataset and the size of the container having been shrunk (which depends on the container block size selected by the user and the amount of block padding inside the container due to data granularity) will usually be negligible with respect to the large size of the compressed dataset, as corroborated by many shrinkage tests we conducted. Being this the case, we finally decided to skip the shrinkage step altogether, and directly report the size of the compressed database instead.

1.2.4 Test setup

The tests were performed on the CNAG cluster. It is made of more than 100 compute nodes each one having two Intel Xeon Quad Core 2.93 GHz processors with 48 GB of RAM. It has about 3 PB of network-distributed hard-drive storage mounted as a *Lustre* parallel file system (http://lustre.org/). Inter-node communication is performed via a dedicated Infiniband network, whereas the *Lustre* filesystem is connected to the cluster via a number of standard Gigabit Ethernet connections. In practice, filesystem access through the network turns out to be the computational bottleneck for many applications.

1.2.5 Results

Volume compression results

In this section we present the results of our large-scale benchmarks on the compression of SAM format. In the tables below the **SAM ratio** column is computed as *original SAM size / compressed size*, while **SAM fraction (%)** is the ratio in percents between the *compressed size* and the *original SAM size*, that is 100 * *compressed size / original SAM size* (ratio and fraction are two common measures used to quantify compression). Analogous definitions hold true for **BAM ratio**, **BAM frac.(%)** and **CRAM frac.(%)**.

Table 1.4: Summary of SAM compression for the small volume

Format	size (GB)	SAM ratio	BAM ratio	SAM frac.(%)	BAM frac.(%)	CRAM frac.(%)
SAM	880.93	—	—	—	—	—
BAM	209.63	4.20	—	23.80	—	—
BAM-Q8	145.00	6.08	1.45	16.46	69.17	—
CRAM	85.30	10.33	2.46	9.68	40.69	—
CARGO	72.91	12.08	2.88	8.28	34.78	85.48

Table 1.5: Summary of SAM compression for the large volume

Format	size (TB)	SAM ratio	BAM ratio	SAM frac.(%)	BAM frac.(%)	CRAM frac.(%)
SAM	17.09	—	—	—	—	—
BAM	4.03	4.24	—	23.58	—	—
BAM-Q8	2.70	6.34	1.49	15.77	66.91	—
CRAM	1.63	10.47	2.47	9.56	40.53	—
CARGO	1.44	11.91	2.81	8.40	35.63	87.90

SAM fields compression results

In this section we break down the global compression results shown in the previous section and show in detail how the *CARGO* method considered in our benchmark is able to compress various sets of SAM fields considered as separated entities. More in detail, in the following table we report information for the following (sets of) SAM fields:

- *Read names*: the contents of the `QNAME` SAM field
- *Quality scores*: the contents of the `QUAL` SAM field
- *Optional fields*: the contents of all the SAM optional fields excluding the `MD` tag (which in our implementation is compressed together with the *Mapping data* below)
- *Mapping data*: the content of all the remaining fields.

In the tables below **SAM fraction (%)** is the ratio in percents between the *size of the information contained in the SAM field* and the *total size of the SAM file*, that is 100 * *size of the information contained in the SAM field / total size of the SAM file*; a similar definition holds true for **CARGO frac.(%)**. **CARGO ratio** is computed as *size of the*

information contained in the SAM field / compressed size of the corresponding ‘CARGO streams collection‘. All sizes are in GB.

Table 1.6: Summary of SAM fields compression for the small volume

Fields	SAM size	SAM frac.(%)	CARGO size	CARGO frac.(%)	CARGO ratio
Read names	35.5	4.0	7.7	10.2	4.59
Mapping data	260.5	29.6	17.3	22.8	15.07
Quality scores	197.3	22.4	39.8	52.4	4.96
Optional fields	387.6	44.0	11.1	14.7	34.77

Table 1.7: Summary of SAM fields compression for the large volume

Fields	SAM size	SAM frac.(%)	CARGO size	CARGO frac.(%)	CARGO ratio
Read names	695.9	4.1	170.3	11.2	4.09
Mapping data	5041.1	29.5	328.7	21.7	15.34
Quality scores	3803.4	22.2	785.5	51.8	4.84
Optional fields	7553.8	44.2	231.0	15.2	32.70

SAM volumes query results

In this section we perform range queries on each considered container (the one compressed with BAM, the one compressed with CRAM, and the one compressed with *CARGO*) and collect time measurements for each query. As *SAMtools* is single-threaded, only one result is reported when *SAMtools* is used to query either the BAM or the CRAM container (in columns **BAM** and **CRAM**, respectively). On the other hand, as *CARGO* can employ a different number of threads to perform the query, several results are reported for it (query performed with 1 thread in column **CARGO-T1**, query performed with 2 threads in column **CARGO-T2**, and so on). Column **Query size** contains the size of the output of the query, in MB. Each table row corresponds to a different query, as described in column **Range/type**. The query will span a `chr:pos_begin - chr:pos_end` range where both `chr` and `pos_begin` have been randomly selected; the range size is 1k nt for the first two rows of each table, 10k nt for rows 3-4, 100k nt for rows 5-6 and 1M nt for rows 7-8. Queries can be either `cold` (when a new range is sampled for the first time) or `hot` (when a query on the same range is repeated and slices of data representing the queried region are likely to be already present in the cache of the filesystem); both timings are reported in order to evaluate the latency of the distributed network file system. Each table entry is an average on 10 runs.

Table 1.8: Summary of range-querying timings for the small volume

Range/type	Query size	BAM	CRAM	CARGO-T1	CARGO-T2	CARGO-T4	CARGO-T8
1k-cold	0.2	0.3	49.5	1.2	1.2	1.2	1.2
1k-hot	0.2	0.2	48.9	1.2	1.2	1.2	1.2
10k-cold	2.3	0.5	50.3	1.9	1.8	2.1	1.8
10k-hot	2.3	0.5	46.2	1.3	1.3	1.3	1.3
100k-cold	27.2	0.8	50.3	2.2	1.7	1.4	1.4
100k-hot	27.2	0.9	49.0	2.2	1.9	1.4	1.4
1M-cold	262.7	2.8	47.0	14.4	5.6	3.5	3.0
1M-hot	262.7	2.4	46.0	10.5	5.6	3.6	3.6

Table 1.9: Summary of range-querying timings for the large volume. SAMtools was unable to query CRAM volume

Range/type	Query size	BAM	CARGO-T1	CARGO-T2	CARGO-T4	CARGO-T8
1k-cold	7.2	0.8	6.6	4.8	4.9	4.6
1k-hot	7.2	0.5	3.2	3.1	3.1	3.0
10k-cold	57.1	2.2	20.0	10.9	10.8	10.0
10k-hot	57.1	1.2	5.4	3.9	3.9	4.1
100k-cold	548.4	7.8	39.4	19.5	14.9	13.2
100k-hot	548.4	5.2	23.2	13.1	8.0	6.9
1M-cold	5607.8	45.1	181.8	92.6	48.1	36.2
1M-hot	5607.8	42.2	176.4	91.9	48.0	39.6

1.3 FASTQ format compression benchmarks

In this section we describe in detail how our FASTQ format compression ratio and throughput benchmarks were performed. We compare the results obtained by a very simple proof-of-concept *CARGO* implementation to those of several state-of-the-art FASTQ format-specific compressors.

1.3.1 Data sets

The single-archive multi-platform test data sets consist of FASTQ files produced by different sequencing platforms: Illumina (*SRR608906_2* and *SRX043656*), Ion Torrent (*ERR039503*) and SOLiD (*SRR445256*). They were downloaded from the Short Reads Archive (SRA) database (see: **Appendix A**). Unfortunately the files for Ion Torrent and SOLiD are relatively small (6 and 5 GB uncompressed, respectively); however, in line with what is usually done in similar benchmarks and in order to achieve a better representation of available technologies, we decided to include them anyway.

In addition, to see how the performance of different solutions scales up with larger archives, we performed a benchmark on a big dataset of *G. gallus* reads (*SRX043656*), sequenced with high coverage on Illumina and consisting of 15 files merged into one large FASTQ file.

1.3.2 Reference solutions

The binaries for reference compressors were downloaded from their official websites or compiled from source with default options set in build scripts.

All applications were tested using 8 processing threads (whenever multi-threading is supported by the application).

All applications were tested in 2 lossless compression configurations: **-FAST* providing a good compression ratio together with fast performance, and **-MAX* providing the highest compression ratio.

GZIP

We used version 2.3.1 of `pigz`, a parallel implementation of the standard `gzip` compression tool (available at http://zlib.net/pigz/).

BZIP2

We used version 1.1.8 of *pbzip2*, a parallel implementation of the *bzip2* compression tool (available at http://compression.ca/pbzip2/).

DSRC

We used version 2.0 of *DSRC* [7] (available at http://sun.aei.polsl.pl/dsrc/).

FQZCOMP

We used version 4.6 of *FQZcomp* [6] (available at http://sourceforge.net/projects/fqzcomp/).

QUIP

We used version 1.1.7 of *Quip* [8] (available at http://homes.cs.washington.edu/~dcjones/quip/).

1.3.3 *CARGO*

We also tested a relatively simple-minded *CARGO* implementations of the FASTQ format. The source code is available in the standard *CARGO* distribution in the directory `cargo/examples/fastq/fastq-format`; together with it, a specialized build script `build.sh` to generate the test binaries. To build and test all the executables (on per set of compression methods chosen for the FASTQ fields, see: *CARGO methods* subsection) one needs the following tools: `cargo_translate` (generates C++ files from record definition in *CARGO* meta-language) and `cargo_tool` (allows container management). They are available in the directory `cargo/tools/`.

The examples were all compiled from source and tested using as runtime parameters 8 processing threads and an 8 MB (in the case of the *-FAST compressors, see *CARGO methods* subsection) or 64 MB (in the case of the *-BEST compressors) block for the input file buffer.

A more detailed description about how the examples were implemented, how they can be compiled step-by-step and which command-line parameters should be used with them is available in the **Supplementary Documentation**.

Container configuration

Before each test, a temporary container was created, setting the available compressible storage space up to a size of 9.1 GB (for details about the creation of the container see **Appendix B**).

1.3.4 Size measurements

The space occupancy results reported for *CARGO* are those provided by `cargo_tool` about the cumulative size of the compressed dataset. As containers are usually arbitrarily big and can contain more than a single dataset, this might be considered as an indirect measurement of the size of the dataset. However, containers can be shrunk to eliminate free blocks and thus reclaim unused space. In the current implementation, the difference between the actual size of the compressed dataset and the size of the container having been shrunk (which depends on the container block size selected by the user and the amount of block padding inside the container due to data granularity) will usually be negligible with respect to the large size of the compressed dataset, as corroborated by many shrinkage tests we conducted. Being this the case, we finally decided to skip the shrinkage step altogether, and directly report the size of the compressed database instead.

CARGO methods

The names of the tested *CARGO* methods follow the pattern:

```
CARGO-<comp_method>-<option>
```

where `comp_method` specifies the compression method name (one of *GZIP*, *BZIP*, *PPMD*, *LZMA* and *OPT*) and `option` specifies the compression method option (either *FAST*, a fast compression setup offering a lower compression ratio, or *MAX*, a slower compression setup offering the maximum compression ratio). The *FAST* method uses compression level 1 for each algorithm, whereas *BEST* uses compression level 4 (see **Supplementary Documentation** for a precise definition of compression levels and methods). The *OPT* compression methods uses different combinations of compression methods for each of the FASTQ fields. In addition, all solutions use 2 MB (default) for an internal compression buffer. Finally, compressors with the *FAST* option use 8 MB, whereas those with *MAX* and *OPT* options use 64 MB as the input file buffer block size.

1.3.5 Test setup

The experiments were performed on a server machine equipped with four 8-core AMD OpteronTM 6136 2.4GHz CPUs, 128 GB of RAM and a RAID-5 disk matrix containing 6 non-solid state HDDs.

1.3.6 Results

Multi-format single-volume compression results

In this subsection we present the results of our benchmarks on the compression of FASTQ format obtained from different sequencing platforms. In the tables below the **Ratio** column is computed as *original FASTQ size / compressed size*, while **C/size** is the size of the compressed dataset in MB. The **C/speed** and **D/speed** fields represent compression and decompression speed in MB/s computed as the *original (or decompressed) FASTQ size / (de)compression time*, while **C/time** and **D/time** are the total compression/decompression processing times in seconds.

It's worth noting that in the case of the *ERR039503* data set and for the majority of the used tools (marked with italic font), the size of decompressed file differs from the original one, as the extra information in the control '+' line is being discarded in the output. This information is present in the original file but is redundant, being a duplication of the read identifier — storing it twice is considered as bad practice (although it is FASTQ format compliant). The decompressed file stripped of the repeated information present in the '+' field has a size reduced by 587.6 MB (10% of the total original file size).

In **Fig. 1.2** we show how our *CARGO*-generated solutions and other FASTQ format-specific compressors perform in terms of both (de)compression speed and ratio.

Table 1.10: Compressing file SRR608906_2 (sample Illumina reads from the SRA) with several methods. Initial FASTQ file size: 12.2 GB

Method	Ratio	C/size	C/speed	D/speed	C/time	D/time
FQZCOMP-MAX	4.98	2437	7	8	1664	1578
QUIP-MAX	4.66	2607	13	13	911	926
QUIP-FAST	4.61	2633	21	16	580	759
DSRC2-MAX	4.39	2770	66	69	183	175
FQZCOMP-FAST	4.38	2774	48	34	253	357
CARGO-PPMD-MAX	4.06	2991	48	43	253	285
CARGO-OPT-PPP	4.06	2992	94	79	129	153
DSRC2-FAST	3.92	3098	267	370	45	32
CARGO-PPMD-FAST	3.89	3127	121	97	100	124
CARGO-LZMA-MAX	3.89	3126	8	196	1523	61
CARGO-OPT-GPP	3.89	3125	84	94	144	128
CARGO-OPT-PLL	3.83	3173	10	150	1240	80
CARGO-LZMA-FAST	3.76	3231	11	201	1086	60
CARGO-BZIP2-MAX	3.70	3283	42	119	287	101
CARGO-BZIP2-FAST	3.64	3342	55	161	219	75
BZIP2-MAX	3.53	3446	47	100	257	120
BZIP2-FAST	3.36	3615	57	185	212	65
CARGO-GZIP-MAX	3.35	3624	16	414	778	29
CARGO-GZIP-FAST	2.94	4126	259	503	46	24
GZIP-MAX	2.89	4198	28	132	430	91
GZIP-FAST	2.53	4802	200	118	60	102

Table 1.11: Compressing file ERR039503 (sample Ion Torrent reads from the SRA) with several methods. Initial FASTQ file size: 6.0 GB

Method	Ratio	C/size	C/speed	D/speed	C/time	D/time
FQZCOMP-MAX	5.58	1069	7	6	900	906
DSRC2-MAX	5.15	1157	76	76	73	78
FQZCOMP-FAST	5.14	1160	35	31	127	171
CARGO-PPMD-MAX	4.84	1231	62	56	85	96
CARGO-OPT-PPP	4.80	1242	94	85	53	63
QUIP-MAX	4.80	1241	9	8	813	655
QUIP-FAST	4.80	1241	16	14	332	381
CARGO-OPT-GPP	4.62	1290	103	93	67	58
CARGO-PPMD-FAST	4.53	1315	109	100	44	54
CARGO-OPT-PLL	4.35	1369	210	192	737	28
CARGO-LZMA-MAX	4.34	1375	238	215	836	25
DSRC2-FAST	4.34	1373	452	459	17	13
CARGO-BZIP2-MAX	4.33	1378	128	117	98	46
CARGO-BZIP2-FAST	4.17	1430	184	168	82	32
CARGO-LZMA-FAST	4.16	1434	251	234	535	23
BZIP2-MAX	4.09	1457	163	166	101	36
BZIP2-FAST	3.85	1548	239	248	104	24
CARGO-GZIP-MAX	3.79	1574	538	489	531	11
GZIP-MAX	3.26	1828	140	142	372	42
CARGO-GZIP-FAST	3.15	1895	581	537	25	10
GZIP-FAST	2.75	2165	115	117	31	51

Table 1.12: Compressing file SRR445256 (sample SOLiD reads from the SRA) with several methods. Initial FASTQ file size: 5.0 GB

Method	Ratio	C/size	C/speed	D/speed	C/time	D/time
FQZCOMP-MAX	5.54	847	13	12	361	382
FQZCOMP-FAST	5.06	928	53	38	89	122
DSRC2-MAX	5.05	929	66	70	70	67
DSRC2-FAST	4.49	1046	242	417	19	11
CARGO-LZMA-MAX	4.46	1054	8	193	587	24
CARGO-OPT-PLL	4.36	1076	15	133	303	35
CARGO-OPT-PPP	4.28	1096	75	66	62	71
CARGO-PPMD-MAX	4.26	1102	41	37	115	127
CARGO-LZMA-FAST	4.13	1137	18	206	264	22
CARGO-BZIP2-MAX	4.13	1136	34	132	138	35
CARGO-OPT-GPP	4.04	1163	71	81	66	58
CARGO-BZIP2-FAST	4.00	1174	49	177	96	26
CARGO-PPMD-FAST	3.94	1190	125	104	37	45
BZIP2-MAX	3.90	1203	37	185	127	25
CARGO-GZIP-MAX	3.75	1250	35	469	135	10
BZIP2-FAST	3.71	1267	50	253	94	18
CARGO-GZIP-FAST	3.33	1410	215	535	21	8
GZIP-MAX	3.25	1445	62	155	75	30
GZIP-FAST	2.80	1679	283	129	16	36

Large-scale compression results

In this subsection we present the results of our benchmarks on the compression of FASTQ format performed on a large data set of *G. gallus* reads (experiment name: *SRX043656*) sequenced on the Illumina platform. As in the previous subsection, in the tables below the **Ratio** column is computed as *original FASTQ size / compressed size*, while **C/size** is the size of the compressed dataset in MB. The **C/speed** and **D/speed** fields represent compression and decompression speed in MB/s computed as the *original (or decompressed) FASTQ size / (de)compression time*, while **C/time** and **D/time** are the total compression/decompression processing times in seconds.

Here too it is worth noting that for the majority of the used tools (marked with italic font), the size of decompressed file differs from the original one, as the extra information in the control '+' line is being discarded in the output. The decompressed file stripped of this redundant information has a size reduced by 22.2 GB (19% of the total original file size), which is a significant and non-negligible amount.

In **Fig. 1.3** we show how our *CARGO*-generated solutions and other FASTQ format-specific compressors perform in terms of both (de)compression speed and ratio.

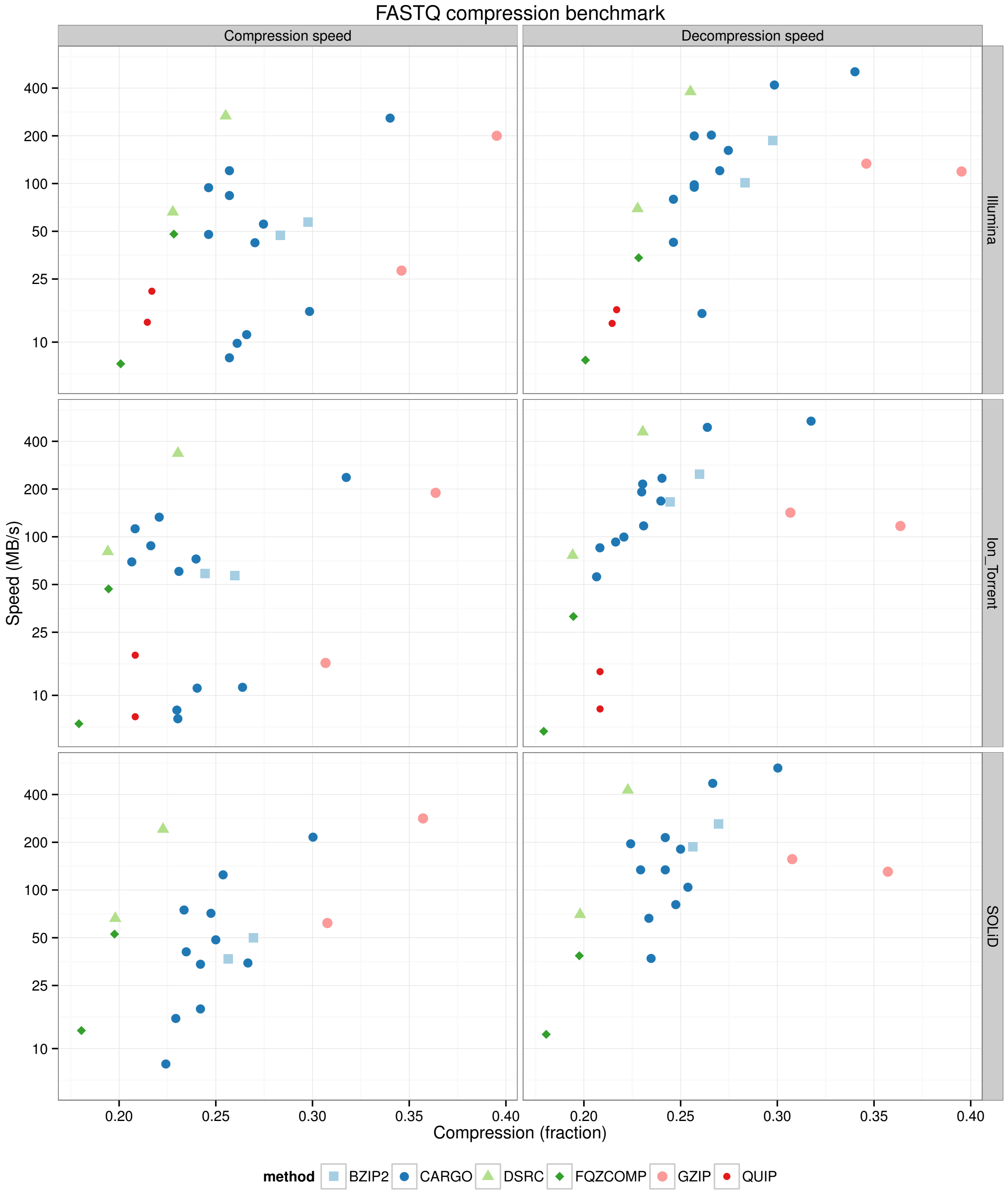

Fig. 1.2: Compression of multi-platform archives in FASTQ format: results of throughput vs. ratio benchmarks

Table 1.13: Compressing the 15 merged archives of SRA data set SRX043656 with several methods. Initial FASTQ file size: 115.9 GB

Method	Ratio	C/size	C/speed	D/speed	C/time	D/time
FQZCOMP-MAX	9.42	12295	10	10	11418	11631
QUIP-MAX	8.46	13691	14	11	8022	10564
QUIP-FAST	8.41	13781	23	11	5131	10111
FQZCOMP-FAST	7.99	14508	48	32	2426	3599
DSRC2-MAX	7.96	14553	94	99	1230	1174
CARGO-PPMD-MAX	7.23	16030	114	98	1013	1185
CARGO-OPT-PPP	7.23	16034	143	119	808	973
DSRC2-FAST	7.20	16082	249	122	464	953
CARGO-LZMA-MAX	6.97	16617	9	252	13265	459
CARGO-OPT-PLL	6.89	16827	10	221	11827	525
CARGO-OPT-GPP	6.82	16980	127	147	910	789
CARGO-PPMD-FAST	6.76	17131	169	134	686	866
CARGO-BZIP2-MAX	6.46	17939	75	185	1547	627
CARGO-LZMA-FAST	6.36	18214	16	268	7183	432
CARGO-BZIP2-FAST	6.34	18276	87	233	1332	498
CARGO-GZIP-MAX	5.90	19650	16	298	7186	388
BZIP2-MAX	5.64	20538	59	103	1960	1123
BZIP2-FAST	5.43	21348	68	109	1710	1061
CARGO-GZIP-FAST	4.80	24128	328	262	353	442
GZIP-MAX	4.66	24838	17	109	6638	1059
GZIP-FAST	3.85	30107	189	100	613	1158

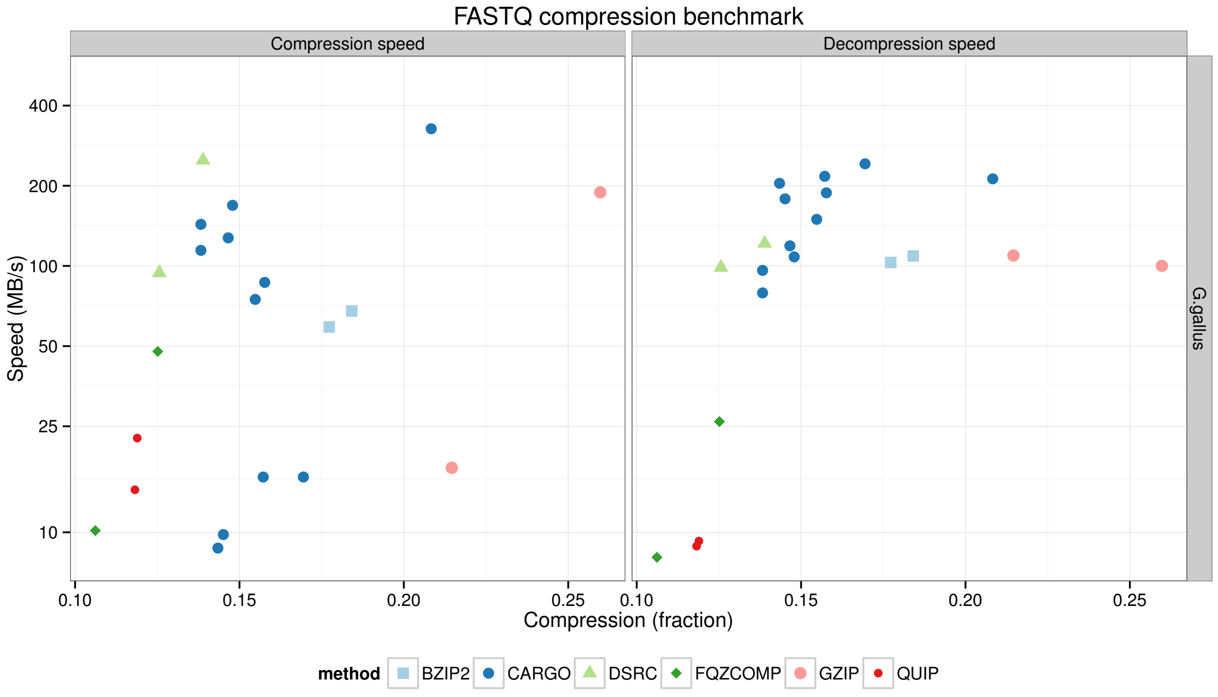

Fig. 1.3: Compression of a large Illumina data set in FASTQ format: results of throughput vs. ratio benchmarks

1.4 Memory and performance scalability of multithreaded CARGO-based compressors

In this section we consider a simple *CARGO*-based FASTQ (de)compressor introduced in the previous section, and examine how performance and memory usage scale up with the number of processing threads, and for different container configurations. As those tests can be very wall-clock time-intensive, we restrict ourselves to single-archive benchmarks of a simple compressor.

1.4.1 Data set

The test data set consists of a single *SRR608906_2* FASTQ file of 12.2 GB in size — the same file was used in the tests presented in the previous section.

1.4.2 *CARGO* solutions

For simplicity and in order to get the results of the tests in a reasonable amount of time, we tested two relatively simple *CARGO* implementations of the FASTQ format compressors that use a *GZIP* compression scheme only. *CARGO-GZIP-LO* is a small-memory (possibly faster, but producing larger output) variant of a *GZIP*-based compressor, using level 1 compression with 256 kB reserved for internal compression buffer and 1 MB reserved for the I/O buffer. In contrast, *CARGO-GZIP-HI* implements a larger-memory (possibly slower, but producing smaller output) variant of a *GZIP*-based compressor – it uses a level 4 compression scheme with 8 MB reserved for internal compression buffer and 64 MB reserved for the I/O buffer.

The source code for these solutions is available in the standard *CARGO* distribution in the directory `cargo/examples/fastq/fastq-benchmark`; a specialized build script `build.sh` can be used to generate the test binaries. To build and test all the executables one needs the following tools: `cargo_translate` (generates C++ files from record definition in *CARGO* meta-language) and `cargo_tool` (allows container management). They are available in the directory `cargo/tools/`.

A more detailed description about how the examples were implemented, how they can be compiled step-by-step and which command-line parameters should be used with them is available in the **Supplementary Documentation**.

Container configuration

Before each test, a temporary container was created, setting the available compressible storage space up to a size of 9.1 GB. Two different container configurations were tested using different combinations of large and small container block sizes, as shown in the table below.

Table 1.14: Used container blocks configurations

Container	Large block size (MiB)	Small block size (KiB)
(1)	1	128
(2)	8	512

For details regarding the creation of the containers and their exact configurations please refer to **Appendix B**.

1.4.3 Performance measurements

The measurements of processing time alongside with memory usage (*Maximum Resident Set Size*) were reported by using the *GNU time* tool while running each solution separately — one for each choice of both the number of processing threads and the container's configuration.

1.4.4 Test setup

The experiments were performed on a server machine equipped with four 8-core AMD OpteronTM 6136 2.4GHz CPUs, 128 GB of RAM and a RAID-5 disk matrix containing 6 non-solid state HDDs.

1.4.5 Results

In **Fig. 1.4** we show how our sample *CARGO*-generated solutions scale in terms of (de)compression speed depending on the number of processing threads used and for different container configurations. **Speed** represents compression and decompression speed in MB/s and is computed as *original or decompressed FASTQ size / (de)compression time*. In **Fig. 1.5** we show how these solutions perform in terms of memory usage for the same test scenarios.

The data used to generate the charts is presented in **Tab. 1.15-1.18**, where the **C/speed** and **D/speed** fields represent compression and decompression speed in MB/s, while **C/time** and **D/time** are the total compression/decompression processing times in seconds. The **C/mem** and **D/mem** fields represent the maximum memory usage both for compression and decompression.

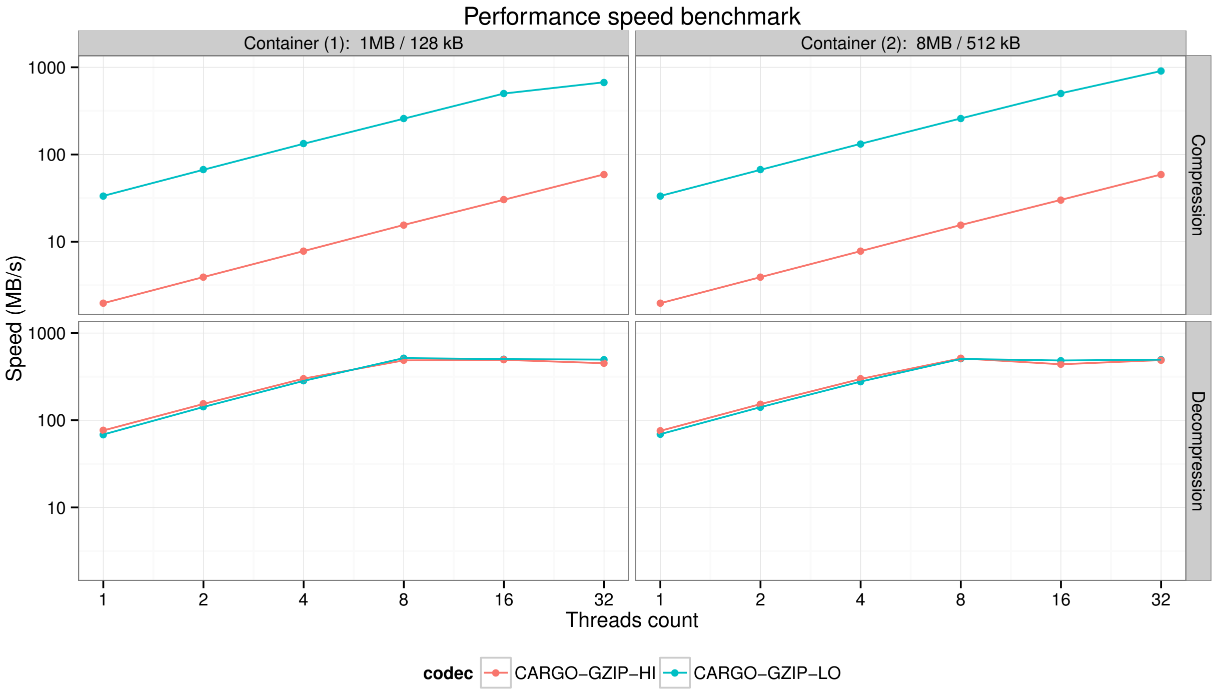

Fig. 1.4: Compression of FASTQ format: throughput scalability for different container configurations

Table 1.15: Compression of FASTQ format: performance and memory usage benchmark results for CARGO-GZIP-LO compressor using container (1)

Threads	C/speed	D/speed	C/time	D/time	C/mem	D/mem
1	33	69	362	177	15	12
2	67	142	181	85	26	26
4	133	283	91	42	53	44
8	257	516	47	23	93	79
16	500	502	24	24	151	150
32	673	496	18	24	351	335

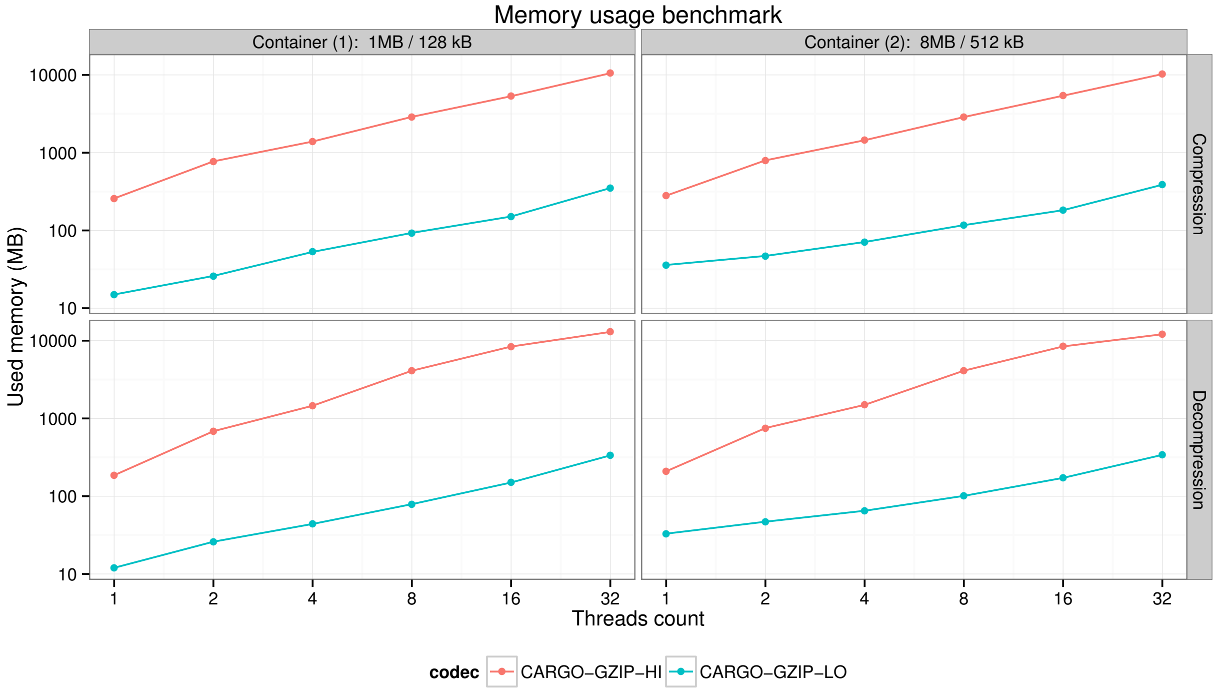

Fig. 1.5: Compression of FASTQ format: maximum memory usage for different container configurations

Table 1.16: Compression of FASTQ format: performance and memory usage benchmark results for CARGO-GZIP-LO compressor using container (2)

Threads	C/speed	D/speed	C/time	D/time	C/mem	D/mem
1	33	69	363	175	36	33
2	67	141	182	85	47	47
4	132	277	91	43	71	65
8	259	505	46	24	117	101
16	503	485	24	25	182	172
32	906	495	13	24	387	340

Table 1.17: Compression of FASTQ format: performance and memory usage benchmark results for CARGO-GZIP-HI compressor using container (1)

Threads	C/speed	D/speed	C/time	D/time	C/mem	D/mem
1	2	76	6182	159	258	186
2	4	154	3094	79	773	685
4	8	300	1557	40	1391	1456
8	16	486	780	25	2888	4105
16	30	493	401	24	5318	8399
32	59	451	205	26	10523	12971

Table 1.18: Compression of FASTQ format: performance and memory usage benchmark results for CARGO-GZIP-HI compressor using container (2)

Threads	C/speed	D/speed	C/time	D/time	C/mem	D/mem
1	2	76	6159	160	280	209
2	4	153	3097	79	796	752
4	8	297	1559	40	1445	1495
8	16	513	782	23	2871	4111
16	30	439	402	27	5402	8435
32	59	490	205	24	10249	12090

1.5 References

[1] BAM format specification. http://samtools.github.io/hts-specs/SAMv1.pdf

[2] CRAM format specification. https://samtools.github.io/hts-specs/CRAMv2.1.pdf

[3] Bonfield JK, *The Scramble conversion tool*. Bioinformatics vol. 30 no. 19, 2014

[4] Illumina, *Reducing Whole-Genome Data Storage Footprint*. Technical report, 2014. http://www.illumina.com/documents/products/whitepapers/whitepaper_datacompression.pdf

[5] Hach I, Faraz S, Cenk S, *DeeZ: reference-based compression by local assembly*. Nature Methods vol. 11, 2014

[6] Bonfield JK, Mahoney MV, *Compression of FASTQ and SAM Format Sequencing Data*. PLoS ONE vol. 8 no. 3, 2013

[7] Roguski L, Deorowicz S, *DSRC 2: Industry-oriented compression of FASTQ files*, Bioinformatics vol. 30 no. 15, 2014

[8] Jones DC, Ruzzo WL, Peng X. Katze MG, *Compression of next-generation sequencing reads aided by highly efficient de novo assembly*. Nucleic Acids Research, 2012

CHAPTER

TWO

APPENDIX A - DATASETS

2.1 SAM format compression benchmarks

Each file in our test data set consists of a number of aligned sequencing reads that come from a different *H. Sapiens* individual; all reads were produced within the 1000 Genomes project. The files in compressed BAM format were downloaded from the project's public `ftp` repository

```
ftp://ftp.ncbi.nlm.nih.gov/1000genomes/ftp/data/
```

Name and size information for all test files in BAM and SAM format (the latter obtained by decompressing the BAM files with *SAMtools* version 1.1) is presented in the table below.

Table 2.1: SAM files data set used in current benchmark

File name	BAM size (GB)	SAM size (GB)
HG00306.mapped.ILLUMINA.bwa.FIN.low_coverage.20120522.sam	15.8	68.3
HG01880.mapped.ILLUMINA.bwa.ACB.low_coverage.20120522.sam	18.4	81.9
HG03780.mapped.ILLUMINA.bwa.ITU.low_coverage.20121211.sam	18.3	75.5

Some of our test scenarios use reference-based compression techniques and thus require the original reference genome to which the sequencing reads have been aligned. In the case of the 1000 Genomes Project the *GRCh37* Human Genome assembly was used. The corresponding `gzip`-compressed FASTA file `hs37d5.fa.gz` was also downloaded from the 1000 Genomes project's public `ftp` repository,

```
ftp://ftp.1000genomes.ebi.ac.uk/vol1/ftp/technical/reference/phase2_reference_assembly_sequence/
```

2.2 Queryable large-scale SAM format benchmarks

The total data for the test consists of 157 BAM files, for a total (compressed) size of 4071 GB. The first collection of files tested (the *small volume*) consists of only 8 BAM files merged into one sorted BAM file; information for such files is provided in the **Table 2.2**. On the other hand, the second collection (the *large volume*) was prepared from all the 157 BAM files downloaded (all the relevant information about them can be found in the **Table 2.3**). Also in this case the files were obtained from the 1000 Genomes project.

Table 2.2: Small volume BAM files

File name	Size (GB)
HG00096.mapped.ILLUMINA.bwa.GBR.low_coverage.20120522.bam	15.6
HG00097.mapped.ILLUMINA.bwa.GBR.low_coverage.20130415.bam	32.0
HG00140.mapped.ILLUMINA.bwa.GBR.low_coverage.20130415.bam	38.1
HG00141.mapped.ILLUMINA.bwa.GBR.low_coverage.20130415.bam	44.2
HG00142.mapped.ILLUMINA.bwa.GBR.low_coverage.20120522.bam	14.1
HG00143.mapped.ILLUMINA.bwa.GBR.low_coverage.20121211.bam	27.3
HG00145.mapped.ILLUMINA.bwa.GBR.low_coverage.20120522.bam	20.2
HG00146.mapped.ILLUMINA.bwa.GBR.low_coverage.20120522.bam	17.3
**Total**	**208.9**

Table 2.3: Large volume BAM files

File name	Size (GB)
HG00096.mapped.ILLUMINA.bwa.GBR.low_coverage.20120522.bam	15.6
HG00097.mapped.ILLUMINA.bwa.GBR.low_coverage.20130415.bam	32.0
HG00099.mapped.ILLUMINA.bwa.GBR.low_coverage.20130415.bam	26.0
HG00100.mapped.ILLUMINA.bwa.GBR.low_coverage.20130415.bam	44.4
HG00101.mapped.ILLUMINA.bwa.GBR.low_coverage.20130415.bam	24.0
HG00102.mapped.ILLUMINA.bwa.GBR.low_coverage.20130415.bam	23.4
HG00103.mapped.ILLUMINA.bwa.GBR.low_coverage.20120522.bam	17.1
HG00105.mapped.ILLUMINA.bwa.GBR.low_coverage.20130415.bam	23.7
HG00106.mapped.ILLUMINA.bwa.GBR.low_coverage.20121211.bam	25.8
HG00107.mapped.ILLUMINA.bwa.GBR.low_coverage.20130415.bam	25.3
HG00108.mapped.ILLUMINA.bwa.GBR.low_coverage.20120522.bam	17.9
HG00109.mapped.ILLUMINA.bwa.GBR.low_coverage.20130415.bam	21.4
HG00110.mapped.ILLUMINA.bwa.GBR.low_coverage.20130415.bam	32.4
HG00111.mapped.ILLUMINA.bwa.GBR.low_coverage.20120522.bam	19.1
HG00112.mapped.ILLUMINA.bwa.GBR.low_coverage.20120522.bam	14.5
HG00113.mapped.ILLUMINA.bwa.GBR.low_coverage.20130415.bam	31.1
HG00114.mapped.ILLUMINA.bwa.GBR.low_coverage.20120522.bam	13.6
HG00115.mapped.ILLUMINA.bwa.GBR.low_coverage.20130415.bam	28.4
HG00116.mapped.ILLUMINA.bwa.GBR.low_coverage.20120522.bam	19.6
HG00117.mapped.ILLUMINA.bwa.GBR.low_coverage.20120522.bam	20.7
HG00118.mapped.ILLUMINA.bwa.GBR.low_coverage.20121211.bam	39.7
HG00119.mapped.ILLUMINA.bwa.GBR.low_coverage.20120522.bam	17.1
HG00120.mapped.ILLUMINA.bwa.GBR.low_coverage.20120522.bam	17.5
HG00121.mapped.ILLUMINA.bwa.GBR.low_coverage.20130415.bam	22.2
HG00122.mapped.ILLUMINA.bwa.GBR.low_coverage.20121211.bam	24.6
HG00123.mapped.ILLUMINA.bwa.GBR.low_coverage.20120522.bam	31.0
HG00124.mapped.ILLUMINA.bwa.GBR.low_coverage.20120522.bam	39.6
HG00125.mapped.ILLUMINA.bwa.GBR.low_coverage.20120522.bam	13.2
HG00126.mapped.ILLUMINA.bwa.GBR.low_coverage.20121211.bam	26.0
HG00127.mapped.ILLUMINA.bwa.GBR.low_coverage.20120522.bam	15.8
HG00128.mapped.ILLUMINA.bwa.GBR.low_coverage.20130415.bam	23.5
HG00129.mapped.ILLUMINA.bwa.GBR.low_coverage.20130415.bam	23.7
HG00130.mapped.ILLUMINA.bwa.GBR.low_coverage.20130415.bam	15.7
HG00131.mapped.ILLUMINA.bwa.GBR.low_coverage.20120522.bam	18.3
HG00132.mapped.ILLUMINA.bwa.GBR.low_coverage.20130415.bam	25.8

Continued on next page

Table 2.3 – continued from previous page

File name	Size (GB)
HG00133.mapped.ILLUMINA.bwa.GBR.low_coverage.20120522.bam	33.0
HG00136.mapped.ILLUMINA.bwa.GBR.low_coverage.20120522.bam	12.6
HG00137.mapped.ILLUMINA.bwa.GBR.low_coverage.20120522.bam	12.7
HG00138.mapped.ILLUMINA.bwa.GBR.low_coverage.20120522.bam	18.3
HG00139.mapped.ILLUMINA.bwa.GBR.low_coverage.20130415.bam	39.5
HG00140.mapped.ILLUMINA.bwa.GBR.low_coverage.20130415.bam	38.1
HG00141.mapped.ILLUMINA.bwa.GBR.low_coverage.20130415.bam	44.2
HG00142.mapped.ILLUMINA.bwa.GBR.low_coverage.20120522.bam	14.1
HG00143.mapped.ILLUMINA.bwa.GBR.low_coverage.20121211.bam	27.3
HG00145.mapped.ILLUMINA.bwa.GBR.low_coverage.20120522.bam	20.2
HG00146.mapped.ILLUMINA.bwa.GBR.low_coverage.20120522.bam	17.3
HG00148.mapped.ILLUMINA.bwa.GBR.low_coverage.20121211.bam	34.2
HG00160.mapped.ILLUMINA.bwa.GBR.low_coverage.20120522.bam	22.0
HG00178.mapped.ILLUMINA.bwa.FIN.low_coverage.20130415.bam	30.0
HG00189.mapped.ILLUMINA.bwa.FIN.low_coverage.20120522.bam	13.5
HG00235.mapped.ILLUMINA.bwa.GBR.low_coverage.20130415.bam	26.9
HG00245.mapped.ILLUMINA.bwa.GBR.low_coverage.20120522.bam	20.7
HG00255.mapped.ILLUMINA.bwa.GBR.low_coverage.20130415.bam	24.0
HG00260.mapped.ILLUMINA.bwa.GBR.low_coverage.20130415.bam	34.9
HG00275.mapped.ILLUMINA.bwa.FIN.low_coverage.20120522.bam	16.8
HG00282.mapped.ILLUMINA.bwa.FIN.low_coverage.20120522.bam	17.4
HG00290.mapped.ILLUMINA.bwa.FIN.low_coverage.20130415.bam	24.4
HG00306.mapped.ILLUMINA.bwa.FIN.low_coverage.20120522.bam	15.8
HG00315.mapped.ILLUMINA.bwa.FIN.low_coverage.20120522.bam	12.4
HG00324.mapped.ILLUMINA.bwa.FIN.low_coverage.20120522.bam	18.8
HG00332.mapped.ILLUMINA.bwa.FIN.low_coverage.20130415.bam	22.2
HG00341.mapped.ILLUMINA.bwa.FIN.low_coverage.20130415.bam	26.5
HG00353.mapped.ILLUMINA.bwa.FIN.low_coverage.20130415.bam	39.2
HG00361.mapped.ILLUMINA.bwa.FIN.low_coverage.20120522.bam	19.7
HG00371.mapped.ILLUMINA.bwa.FIN.low_coverage.20130415.bam	24.3
HG00379.mapped.ILLUMINA.bwa.FIN.low_coverage.20130415.bam	23.3
HG00410.mapped.ILLUMINA.bwa.CHS.low_coverage.20121211.bam	35.0
HG00422.mapped.ILLUMINA.bwa.CHS.low_coverage.20130415.bam	38.3
HG00448.mapped.ILLUMINA.bwa.CHS.low_coverage.20130415.bam	32.6
HG00458.mapped.ILLUMINA.bwa.CHS.low_coverage.20130415.bam	28.8
HG00530.mapped.ILLUMINA.bwa.CHS.low_coverage.20120522.bam	16.1
HG00534.mapped.ILLUMINA.bwa.CHS.low_coverage.20120522.bam	17.3
HG00557.mapped.ILLUMINA.bwa.CHS.low_coverage.20130415.bam	34.8
HG00566.mapped.ILLUMINA.bwa.CHS.low_coverage.20120522.bam	14.3
HG00610.mapped.ILLUMINA.bwa.CHS.low_coverage.20120522.bam	16.9
HG00638.mapped.ILLUMINA.bwa.PUR.low_coverage.20120522.bam	16.1
HG00674.mapped.ILLUMINA.bwa.CHS.low_coverage.20121211.bam	31.0
HG00702.mapped.ILLUMINA.bwa.CHS.low_coverage.20120522.bam	15.1
HG00732.mapped.ILLUMINA.bwa.PUR.low_coverage.20130422.bam	105.8
HG01029.mapped.ILLUMINA.bwa.CDX.low_coverage.20130415.bam	24.4
HG01075.mapped.ILLUMINA.bwa.PUR.low_coverage.20120522.bam	58.0
HG01089.mapped.ILLUMINA.bwa.PUR.low_coverage.20121211.bam	16.3
HG01104.mapped.ILLUMINA.bwa.PUR.low_coverage.20130415.bam	24.4
HG01272.mapped.ILLUMINA.bwa.CLM.low_coverage.20130415.bam	21.4

Continued on next page

Table 2.3 – continued from previous page

File name	Size (GB)
HG01441.mapped.ILLUMINA.bwa.CLM.low_coverage.20120522.bam	21.4
HG01443.mapped.ILLUMINA.bwa.CLM.low_coverage.20121211.bam	18.0
HG01589.mapped.ILLUMINA.bwa.PJL.low_coverage.20130415.bam	17.1
HG01767.mapped.ILLUMINA.bwa.IBS.low_coverage.20130415.bam	26.7
HG01861.mapped.ILLUMINA.bwa.KHV.low_coverage.20130415.bam	23.3
HG01880.mapped.ILLUMINA.bwa.ACB.low_coverage.20120522.bam	18.4
HG02006.mapped.ILLUMINA.bwa.PEL.low_coverage.20130415.bam	25.9
HG02082.mapped.ILLUMINA.bwa.KHV.low_coverage.20130415.bam	28.7
HG02184.mapped.ILLUMINA.bwa.CDX.low_coverage.20120522.bam	15.0
HG02387.mapped.ILLUMINA.bwa.CDX.low_coverage.20120522.bam	18.3
HG02425.mapped.ILLUMINA.bwa.PEL.low_coverage.20130415.bam	20.4
HG02541.mapped.ILLUMINA.bwa.ACB.low_coverage.20130415.bam	22.6
HG02611.mapped.ILLUMINA.bwa.GWD.low_coverage.20121211.bam	39.0
HG02716.mapped.ILLUMINA.bwa.GWD.low_coverage.20121211.bam	33.4
HG02840.mapped.ILLUMINA.bwa.GWD.low_coverage.20121211.bam	20.1
HG03054.mapped.ILLUMINA.bwa.MSL.low_coverage.20130415.bam	30.5
HG03189.mapped.ILLUMINA.bwa.ESN.low_coverage.20130415.bam	21.8
HG03445.mapped.ILLUMINA.bwa.MSL.low_coverage.20121211.bam	26.1
HG03604.mapped.ILLUMINA.bwa.BEB.low_coverage.20130415.bam	20.1
HG03625.mapped.ILLUMINA.bwa.PJL.low_coverage.20130415.bam	30.7
HG03731.mapped.ILLUMINA.bwa.ITU.low_coverage.20130415.bam	36.9
HG03780.mapped.ILLUMINA.bwa.ITU.low_coverage.20121211.bam	18.3
HG03808.mapped.ILLUMINA.bwa.BEB.low_coverage.20121211.bam	18.9
HG03905.mapped.ILLUMINA.bwa.BEB.low_coverage.20121211.bam	17.1
HG04038.mapped.ILLUMINA.bwa.STU.low_coverage.20130415.bam	16.6
HG04185.mapped.ILLUMINA.bwa.BEB.low_coverage.20130415.bam	14.8
HG04239.mapped.ILLUMINA.bwa.ITU.low_coverage.20130415.bam	25.1
NA06984.mapped.ILLUMINA.bwa.CEU.low_coverage.20120522.bam	29.0
NA06985.mapped.ILLUMINA.bwa.CEU.low_coverage.20120522.bam	57.7
NA06986.mapped.ILLUMINA.bwa.CEU.low_coverage.20130415.bam	50.7
NA06989.mapped.ILLUMINA.bwa.CEU.low_coverage.20120522.bam	17.6
NA06994.mapped.ILLUMINA.bwa.CEU.low_coverage.20120522.bam	18.4
NA07000.mapped.ILLUMINA.bwa.CEU.low_coverage.20130415.bam	35.2
NA07037.mapped.ILLUMINA.bwa.CEU.low_coverage.20130502.bam	26.1
NA07048.mapped.ILLUMINA.bwa.CEU.low_coverage.20120522.bam	19.0
NA07051.mapped.ILLUMINA.bwa.CEU.low_coverage.20120522.bam	12.5
NA07056.mapped.ILLUMINA.bwa.CEU.low_coverage.20130415.bam	18.7
NA07347.mapped.ILLUMINA.bwa.CEU.low_coverage.20130415.bam	46.0
NA07357.mapped.ILLUMINA.bwa.CEU.low_coverage.20130415.bam	21.1
NA10847.mapped.ILLUMINA.bwa.CEU.low_coverage.20130502.bam	34.1
NA10851.mapped.ILLUMINA.bwa.CEU.low_coverage.20130415.bam	18.7
NA11829.mapped.ILLUMINA.bwa.CEU.low_coverage.20130415.bam	46.5
NA11830.mapped.ILLUMINA.bwa.CEU.low_coverage.20120522.bam	11.7
NA11831.mapped.ILLUMINA.bwa.CEU.low_coverage.20120522.bam	22.9
NA11832.mapped.ILLUMINA.bwa.CEU.low_coverage.20120522.bam	62.1
NA11840.mapped.ILLUMINA.bwa.CEU.low_coverage.20120522.bam	30.8
NA11843.mapped.ILLUMINA.bwa.CEU.low_coverage.20120522.bam	19.6
NA11881.mapped.ILLUMINA.bwa.CEU.low_coverage.20120522.bam	26.9
NA11892.mapped.ILLUMINA.bwa.CEU.low_coverage.20130415.bam	28.3

Continued on next page

Table 2.3 – continued from previous page

File name	Size (GB)
NA11893.mapped.ILLUMINA.bwa.CEU.low_coverage.20130415.bam	24.0
NA11894.mapped.ILLUMINA.bwa.CEU.low_coverage.20130415.bam	19.3
NA11918.mapped.ILLUMINA.bwa.CEU.low_coverage.20130415.bam	33.6
NA11919.mapped.ILLUMINA.bwa.CEU.low_coverage.20130415.bam	47.5
NA11920.mapped.ILLUMINA.bwa.CEU.low_coverage.20130415.bam	42.9
NA11930.mapped.ILLUMINA.bwa.CEU.low_coverage.20130415.bam	26.7
NA11931.mapped.ILLUMINA.bwa.CEU.low_coverage.20130415.bam	18.8
NA11932.mapped.ILLUMINA.bwa.CEU.low_coverage.20130415.bam	22.1
NA11933.mapped.ILLUMINA.bwa.CEU.low_coverage.20130415.bam	21.9
NA11992.mapped.ILLUMINA.bwa.CEU.low_coverage.20120522.bam	30.4
NA11994.mapped.ILLUMINA.bwa.CEU.low_coverage.20120522.bam	56.2
NA11995.mapped.ILLUMINA.bwa.CEU.low_coverage.20120522.bam	19.4
NA12003.mapped.ILLUMINA.bwa.CEU.low_coverage.20120522.bam	21.4
NA12004.mapped.ILLUMINA.bwa.CEU.low_coverage.20121211.bam	36.3
NA12005.mapped.ILLUMINA.bwa.CEU.low_coverage.20120522.bam	45.6
NA18548.mapped.ILLUMINA.bwa.CHB.low_coverage.20130415.bam	23.3
NA18747.mapped.ILLUMINA.bwa.CHB.low_coverage.20130415.bam	29.9
NA18881.mapped.ILLUMINA.bwa.YRI.low_coverage.20130415.bam	19.7
NA19005.mapped.ILLUMINA.bwa.JPT.low_coverage.20120522.bam	27.5
NA19735.mapped.ILLUMINA.bwa.MXL.low_coverage.20130415.bam	22.5
NA20509.mapped.ILLUMINA.bwa.TSI.low_coverage.20130415.bam	23.0
NA20773.mapped.ILLUMINA.bwa.TSI.low_coverage.20130415.bam	18.0
NA20895.mapped.ILLUMINA.bwa.GIH.low_coverage.20120522.bam	24.6
NA21115.mapped.ILLUMINA.bwa.GIH.low_coverage.20130415.bam	18.0
**Total**	**4071.6**

2.3 FASTQ format compression benchmarks

2.3.1 Multi-format single-volume data set

In this case, the data set for each test we performed consists of a single FASTQ file containing reads generated by a specific sequencing platform (we ran tests on Illumina, Ion Torrent and SOLiD reads). The test FASTQ files were downloaded in `gzip` compressed format from the Short Read Archive (SRA) public `ftp` repository. In more detail, the Illumina and SOLiD data sets were downloaded from

```
http://ftp.sra.ebi.ac.uk/vol1/fastq/
```

while Ion Torrent reads were obtained from:

```
ftp://ftp-trace.ncbi.nlm.nih.gov/sra/sra-instant/reads/ByRun/sra/
```

Information about name, size, originating organism, sequencing protocol and read lengths is presented for each test file in the following table.

Table 2.4: FASTQ files used in the benchmark

File name	Format	Organism	Strategy	Size (MB)	Seq. length
SRR608906_2.fastq	Illumina	A. mexicanus	WGS	12151	100
ERR039503.fastq	Ion Torrent	H. sapiens	WGS	5962	5 - 2716
SRR445256.fastq	SOLiD	B. anthracis	RNA-Seq	4696	51

2.3.2 Large-scale data set

The data set with accession name `SRX043656` comprises 15 FASTQ files. The test FASTQ files were downloaded in `gzip` compressed format from the Short Read Archive (SRA) public `ftp` repository :

```
ftp://ftp.ddbj.nig.ac.jp/ddbj_database/dra/fastq/SRA030/SRA030308/SRX043656/
```

After decompression, the files were merged into one large FASTQ file of size 115.9 GB. Read length is 100 nucleotides.

CHAPTER

THREE

APPENDIX B - TOOLS INVOCATION

3.1 SAM format compression benchmarks

3.1.1 Reference compressors

Whenever the compressor supports multi-threading, it was tested using 8 processing threads.

GZIP

- *GZIP-FAST*

  compress:

```
pigz --fast --processes 8 --stdout IN.sam > OUT.gz
```

  decompress:

```
pigz -d --processes 8 --stdout IN.gz > OUT.sam
```

- *GZIP-BEST*

  compress:

```
pigz --best --processes 8 --stdout IN.sam > OUT.gz
```

  decompress:

```
pigz -d --processes 8 --stdout IN.gz > OUT.sam
```

BZIP2

- *BZIP2-FAST*

  compress:

```
pbzip2 -1 -p8 --stdout IN.sam > OUT.bz2
```

  decompress:

```
pbzip2 -d -p8 --stdout IN.bz2 > OUT.sam
```

- *BZIP2-BEST*

  compress:

```
pbzip2 -9 -p8 --stdout IN.sam > OUT.bz2
```

decompress:

```
pbzip2 -d -p8 --stdout IN.bz2 > OUT.sam
```

SAMTOOLS

- *SAMTOOLS-BAM*

  compress:

```
samtools view -b -@ 8 IN.sam > OUT.bam
```

  decompress:

```
samtools view -h -@ 8 IN.bam > OUT.sam
```

- *SAMTOOLS-CRAM*

  compress:

```
samtools view -C -T REF.fasta -@ 8 IN.sam > OUT.cram
```

  decompress:

```
samtools view -h -T REF.fasta -@ 8 IN.cram > OUT.sam
```

SCRAMBLE

- *SCRAMBLE-BAM*

  compress:

```
scramble -I sam -O bam -m -t 8 IN.sam > OUT.bam
```

  decompress:

```
scramble -I bam -O sam -m -t 8 IN.bam > OUT.sam
```

- *SCRAMBLE-CRAM*

  compress:

```
scramble -I sam -O cram -m -r REF.fasta -t 8 IN.sam > OUT.cram
```

  decompress:

```
scramble -I cram -O sam -m -r REF.fasta -t 8 IN.cram > OUT.sam
```

- *SCRAMBLE-CRAM-Q8*

  compress:

```
scramble -I sam -O cram -m -r REF.fasta -B -t 8 IN.sam > OUT.cram
```

  decompress:

```
scramble -I cram -O sam -m -r REF.fasta -t 8 IN.cram > OUT.sam
```

DEEZ

- *DEEZ-NORMAL*

  compress:

```
deez -t 8 -r REF.fasta IN.sam -c > OUT.dz
```

  decompress:

```
deez -t 8 -r REF.fasta IN.dz -c > OUT.sam
```

- *DEEZ-SAMCOMP*

  compress:

```
deez -t 8 -r REF.fasta -q1 IN.sam -c > OUT.dz
```

  decompress:

```
deez -t 8 -r REF.fasta IN.dz -c > OUT.sam
```

- *DEEZ-Q8*

  compress:

```
deez -t 8 -r REF.fasta -l30 IN.sam -c > OUT.dz
```

  decompress:

```
deez -t 8 -r REF.fasta IN.dz -c > OUT.sam
```

3.1.2 *CARGO* container configuration

Before each test, we created a temporary container using 2048 (`32` * 64) large blocks of `8` MiB each and 1024 (`16` * 64) small blocks of `512` KiB each, sufficient to store compressed data up to a size of

```
2048 * 8 MiB + 1024 * 512 KiB = 16.5 GiB (17 716 740 096 B)
```

where

```
1 KiB = 1024 B
1 MiB = 1024 KiB = 1024 * 1024 B
...
```

The following command line was used in order to create such a container:

```
cargo_tool --create-container --container-file=CONTAINER \
    --large-block-size=8 --large-block-count=32 \
    --small-block-size=512 --small-block-count=16
```

The space available in the container was sufficient for all the tested SAM files. More information about the container architecture concept, the command-line description for `cargo_tool` and examples of use can be found in the **Supplementary Documentation**.

3.1.3 *CARGO* methods

Each *CARGO* method was tested using 8 processing threads and an input buffer size of 64 MiB.

CARGO-SAM-STD

- *CARGO-SAM-STD*:

  compress:

```
cargo_samrecord_toolkit-std c -c CONTAINER -n DATASET -i IN.sam -t 8 -b 64
```

  decompress:

```
cargo_samrecord_toolkit-std d -c CONTAINER -n DATASET -o OUT.sam -t 8
```

- *CARGO-SAM-STD-Q8*

  compress:

```
cargo_samrecord_toolkit-std c -c CONTAINER -n DATASET -i IN.sam -a -t 8 -b 64
```

  decompress:

```
cargo_samrecord_toolkit-std d -c CONTAINER -n DATASET -o OUT.sam -t 8
```

CARGO-SAM-EXT

- *CARGO-SAM-EXT*

  compress:

```
cargo_samrecord_toolkit-ext c -c CONTAINER -n DATASET -t 8 -b 64 \
    -i IN.sam
```

  decompress:

```
cargo_samrecord_toolkit-ext d -c CONTAINER -n DATASET -t 8 \
    -o OUT.sam
```

- *CARGO-SAM-EXT-Q8*

  compress:

```
cargo_samrecord_toolkit-ext c -c CONTAINER -n DATASET -a -t 8 -b 64 \
    -i IN.sam
```

  decompress:

```
cargo_samrecord_toolkit-ext d -c CONTAINER -n DATASET -t 8 \
    -o OUT.sam
```

CARGO-SAM-REF

Before running the actual tests, we turned the *GRCh37* build of the reference genome for *H.sapiens* in FASTA format (see: **Appendix A**) into a compressed and searchable file (see: **CARGO documentation**). This binary representation, that we named *BFF (Binary Fasta File)* allows for fast subsequent sequence queries into the reference. The BFF file was created by running

```
cargo_samrecord_toolkit-ref r -i REF.fasta -o REF.bff
```

Having generated the index for the reference file, the following command lines were used to perform the tests:

- *CARGO-SAM-REF*

compress:

```
cargo_samrecord_toolkit-ref c -c CONTAINER -n DATASET -t 8 -b 64 -a \
    -i IN.sam
```

decompress:

```
cargo_samrecord_toolkit-ref d -c CONTAINER -n DATASET -t 8 -a \
    -f REF.bff -o OUT.sam
```

- *CARGO-SAM-REF-Q8*

  compress:

```
cargo_samrecord_toolkit-ref-q8 c -c CONTAINER -n DATASET -t 8 -b 64 -a \
    -i IN.sam
```

  decompress:

```
cargo_samrecord_toolkit-ref-q8 d -c CONTAINER -n DATASET -t 8 -a \
    -f REF.bff -o OUT.sam
```

- *CARGO-SAM-REF-Q8-MAX*

  compress:

```
cargo_samrecord_toolkit-ref-q8-max c -c CONTAINER -n DATASET -t 8 -b 64 -a \
    -i IN.sam
```

  decompress:

```
cargo_samrecord_toolkit-ref-q8-max d -c CONTAINER -n DATASET -t 8 -a \
    -f REF.bff -o OUT.sam
```

3.1.4 *CARGO* compressed dataset size measurements

To report the size of the compressed dataset stored inside container in addition with information about records count, stream sizes and used blocks number, the *CARGO*-generated compressor applications should be run with extra verbose flag `-v`.

In any case, the reported information about compressed dataset stored inside container can be always retrieved by using `cargo_tool`:

```
cargo_tool --print-dataset --container-file=CONTAINER --dataset-name=DATASET
```

3.2 Queryable large-scale SAM format benchmarks

3.2.1 Volumes preparation

To prepare the data for both the *small* and the *large volume*, we used *SAMtools* and *sCRAMble* (with multi-threaded decompression support) and the *CARGO-SAM-REF-Q8* method, as explained in *Queryable large-scale SAM format benchmarks* section of **Supplementary data**.

In the first step, we merged the (already sorted) BAM files into a single large sorted BAM file:

```
samtools merge -@ 8 SAM_VOLUME.bam IN1.bam IN2.bam ...
```

Having the SAM_VOLUME.bam generated, in the next step we set to apply the Illumina Q-scores reduction scheme, saving the output as SAM_VOLUME_Q8.bam:

```
scramble -I bam -O bam -t 8 -B SAM_VOLUME.bam SAM_VOLUME_Q8.bam
```

In parallel, we performed a BAM-to-CRAM format conversion with applied Illumina Q-scores reduction:

```
scramble -I bam -O cram -r REF.fa -t 8 -B SAM_VOLUME.bam SAM_VOLUME_Q8.cram
```

Finally, we compressed the sorted SAM_VOLUME.bam into a dataset named SAM, and stored it into the *CARGO* container CARGO_VOLUME:

```
scramble -I bam -O sam -t 6 | cargo_samrecord_toolkit-ref-q8-max c -c CARGO_VOLUME \
    -n SAM -t 6 -b 64 -a -g -z
```

Having the volumes generated in BAM and CRAM formats, we indexed them in order to be able to perform subsequent range queries with *SAMtools*:

```
samtools index SAM_VOLUME.bam
samtools index SAM_VOLUME_Q8.bam
samtools index SAM_VOLUME_Q8.cram
```

In the case of *CARGO* methods, the generated record blocks inside the container were transparently indexed during compression thanks to option -g.

CARGO containers configuration

Before running the tests, we created containers able to accommodate the compressed data produced by the selected *CARGO-SAM-REF-Q8* compressor. In order to do so, we decided to set the container size to the 10% of the uncompressed input data. Hence we created an empty container of ~86 GB for the *small volume* test, and an empty container of 1.72 TB for the *large volume* test.

Small volume

We created a temporary container using 10240 (160 * 64) large blocks of 8 MiB each and 1024 (16 * 64) small blocks of 512 KiB each, containing enough empty space to store compressed data up to a size of

```
10240 * 8 MiB + 1024 * 512 KiB = 80.5 GiB (86 436 216 832 B)
```

The following command line was used in order to create such a container:

```
cargo_tool --create-container --container-file=CARGO_VOLUME \
    --large-block-size=8 --large-block-count=160 \
    --small-block-size=512 --small-block-count=16
```

Large volume

We created a temporary container using 204800 (3200 * 64) large blocks of 8 MiB each and 1024 (16 * 64) small blocks of 512 KiB each, sufficient to store compressed data up to a size of

```
204800 * 8 MiB + 1024 * 512 KiB = 1.56 TiB (1 718 523 789 312 B)
```

The following command line was used in order to create such a container:

```
cargo_tool --create-container --container-file=CARGO_VOLUME \
    --large-block-size=8 --large-block-count=3200 \
    --small-block-size=512 --small-block-count=16
```

More information about the container architecture concept, the command-line description for `cargo_tool` and examples of use can be found in the **Supplementary Documentation**.

3.2.2 Optional volumes shrinkage

In order to optimize space usage, a container can be optionally shrank to fit its size to the size of the contained data. This operation removes free and unoccupied blocks and pads the number of the available blocks to the nearest multiple of *64* (for more information see the **Supplementary Documentation**). To shrink the volume one should say

```
cargo_tool --shrink-container --container-file=CARGO_VOLUME
```

In our test cases the *small volume* was shrunk to 73.1 GB, corresponding to the following blocks configuration:

```
8704 * 8 MiB + 192 * 512 KiB = 68.1 GiB (73 115 107 328 B)
```

whereas the *large volume* was shrunk to 1.44 TB, corresponding to the following blocks configuration:

```
171200 * 8 MiB + 192 * 512 KiB = 1.31 GiB (1 436 230 352 896 B)
```

Apart from a tiny overhead, those sizes essentially coincide with the corrsponding total sizes of the underlying compressed streams (72.9 GB and 1.44 TB, respectively).

Of note, `cargo_tool` provides a command to display the information about the container size configuration including how many blocks were allocated:

```
cargo_tool --print-blocks --container-file=CARGO_VOLUME
```

3.2.3 Querying

- BAM:

```
samtools view SAM_VOLUME_Q8.bam KEY > OUT.sam
```

- CRAM:

```
samtools view SAM_VOLUME_Q8.cram -T REF.fa KEY > OUT.sam
```

- CARGO:

```
cargo_samrecord_toolkit-ref-q8 e -c CARGO_VOLUME -n SAM \
    -f REF.bff -t TH -k KEY -a -g -z > OUT.sam
```

where `TH` specifies the number of decompressing threads used when querying for data and `KEY` specifies the query range, as follows:

- BAM / CRAM:

```
chrom:pos_begin-pos_end
```

- CARGO:

```
chrom:pos_begin::chrom:pos_end
```

For example, to extract from `SAM_VOLUME.bam` all the SAM records belonging to chromosome `2` in the range from position `20,100,000` to position `20,200,000` with *SAMtools* one should say

```
samtools view SAM_VOLUME.bam 2:20,100,000-20,200,000 > OUT.sam
```

while the equivalent *CARGO* command (extracting from the SAM dataset contained in CARGO_VOLUME and using 2 extraction threads) would be

```
cargo_samrecord_toolkit-ref-q8 e -c CARGO_VOLUME -n SAM \
    -f REF.bff -t 2 -k 2:20100000::2:20200000 -a -g -z > OUT.sam
```

3.3 FASTQ format compression benchmarks

3.3.1 Reference compressors

GZIP

- *GZIP-FAST*

  compress:

```
        pigz --fast --processes 8 --stdout IN.fastq > OUT.gz
```

  decompress:

```
        pigz -d --processes 8 --stdout IN.gz > OUT.fastq
```

- *GZIP-BEST*

  compress:

```
        pigz --best --processes 8 --stdout IN.fastq > OUT.gz
```

  decompress:

```
        pigz -d --processes 8 --stdout IN.gz > OUT.fastq
```

BZIP2

- *BZIP2-FAST*

  compress:

```
        pbzip2 -1 -p8 --stdout IN.fastq > OUT.bz2
```

  decompress:

```
        pbzip2 -d -p8 --stdout IN.bz2 > OUT.fastq
```

- *BZIP2-BEST*

  compress:

```
        pbzip2 -9 -p8 --stdout IN.fastq > OUT.bz2
```

  decompress:

```
        pbzip2 -d -p8 --stdout IN.bz2 > OUT.fastq
```

DSRC2

- *DSRC2-FAST*

  compress:

```
dsrc c -m0 -t 8 IN.fastq OUT.dsrc
```

  decompress:

```
dsrc d -t 8 IN.dsrc OUT.fastq
```

- *DSRC2-BEST*

  compress:

```
dsrc c -m2 -t 8 IN.fastq OUT.dsrc
```

  decompress:

```
dsrc d -t 8 IN.dsrc OUT.fastq
```

FQZCOMP

- *FQZCOMP-FAST*

  compress:

```
fqz_comp c -n1 -s1 -q1 IN.fastq OUT.fqz
```

  decompress:

```
fqz_comp -d IN.fqz OUT.fastq
```

- *FQZCOMP-BEST*

  compress:

```
fqz_comp -n2 -q3 -s8+ -b IN.fastq OUT.fqz
```

  decompress:

```
fqz_comp -d IN.fqz OUT.fastq
```

QUIP

- *QUIP-FAST*

  compress:

```
quip -c IN.fastq > OUT.qp
```

  decompress:

```
quip -d -c IN.qp OUT.fastq
```

- *QUIP-BEST*

  compress:

```
quip -c IN.fastq OUT.qp
```

decompress:

```
quip -d -c IN.qp OUT.fastq
```

3.3.2 *CARGO* methods

Container configuration

Multi-format single-volume test

Before each test, we created a temporary container using 1024 (16 * 64) large blocks of 8 MiB each and 2048 (32 * 64) small blocks of 256 KiB each, containing enough empty space to store compressed data up to a size of

```
16 * 64 * 8 MiB + 32 * 64 * 256 KiB = 8.5 GiB (9 126 805 504 B)
```

The following command line was used in order to create such a container:

```
cargo_tool --create-container --container-file=CONTAINER \
    --large-block-size=8 --large-block-count=16 \
    --small-block-size=256 --small-block-count=32
```

The space available in the container was sufficient for all the tested FASTQ files. More information about the container architecture concept, the command-line description for cargo_tool and examples of use can be found in the **Supplementary Documentation**.

Large-scale test

In this case we created a temporary container using 4096 (64 * 64) large blocks of 8 MiB each and 2048 (32 * 64) small blocks of 256 KiB each, sufficient to store compressed data up to a size of

```
64 * 64 * 8 MiB + 32 * 64 * 256 KiB = 32.5 GiB (34 896 609 280 B)
```

The following command line was used in order to create such a container:

```
cargo_tool --create-container --container-file=CONTAINER \
    --large-block-size=8 --large-block-count=64 \
    --small-block-size=256 --small-block-count=32
```

The space available in the container was sufficient for all the tested FASTQ files. More information about the container architecture concept, the command-line description for cargo_tool and examples of use can be found in the **Supplementary Documentation**.

Running *CARGO*

In order to compress a FASTQ file into a *CARGO* container one should say

```
cargo_fastqrecord_toolkit-* c -c CONTAINER -n DATASET -t 8 \
    -b BUFFER_SIZE -i IN.fastq
```

where BUFFER_SIZE specifies the size of the input block buffer and DATASET the dataset name under which the FASTQ data will be saved. In addition, in our tests we used an 8 MiB buffer for all the *-*FAST* methods and a 64 MiB buffer for all the *-*BEST* methods.

One can decompress a FASTQ file from a *CARGO* container with the command

```
cargo_fastqrecord_toolkit-* d -c CONTAINER -n DATASET -t 8 \
    -o OUT.fastq
```

where `cargo_fastqrecord_toolkit-*` is any FASTQ compressor/decompressor corresponding to one of the implemented *CARGO* methods.

3.3.3 *CARGO* compressed dataset size measurements

To report the size of the compressed dataset stored inside container in addition with information about records count, stream sizes and used blocks number, the *CARGO*-generated compressor applications should be run with extra verbose flag `-v`.

In any case, the reported information about compressed dataset stored inside container can be always retrieved by using `cargo_tool`:

```
cargo_tool --print-dataset --container-file=CONTAINER --dataset-name=DATASET
```

3.4 Memory and performance scalability of multithreaded CARGO-based compressors

3.4.1 *CARGO* methods

Container configuration

In this set of tests we used 2 different containers configurations, with their parameters specified below.

Table 3.1: Used container blocks configurations – parameters

Container	Large block size	Large block count	Small block size	Small block count
(1)	1	128	128	64
(2)	8	16	512	16

Before running each of the tests, we created a temporary container using `LARGE_BLOCK_COUNT` * 64 large blocks of `LARGE_BLOCK_SIZE` MiB each and `SMALL_BLOCK_COUNT` * 64 small blocks of `SMALL_BLOCK_SIZE` KiB each, sufficient to store compressed data up to a size of 8.5 GiB (9 126 805 504 B).

The following command line was used in order to create such a container:

```
cargo_tool --create-container --container-file=CONTAINER \
    --large-block-size=LARGE_BLOCK_SIZE --large-block-count=LARGE_BLOCK_COUNT \
    --small-block-size=SMALL_BLOCK_SIZE --small-block-count=SMALL_BLOCK_COUNT
```

The space available in the container was sufficient for the tested FASTQ file. More information about the container architecture concept, the command-line description for `cargo_tool` and examples of use can be found in the **Supplementary Documentation**.

Running *CARGO*

In order to compress a FASTQ file into a *CARGO* container one should say

```
cargo_fastqrecord_toolkit-* c -c CONTAINER -n DATASET -t THREADS \
    -b BUFFER_SIZE -i IN.fastq
```

where `THREADS` specifies the number of processing threads, `DATASET` specifies the dataset name under which the FASTQ data will be saved and `BUFFER_SIZE` specifies the size of the input block buffer – we used an `1` MiB buffer in the *CARGO-GZIP-LO* method and a `64` MiB buffer in the *CARGO-GZIP-HI* method.

One can decompress a FASTQ file from a *CARGO* container with the command

```
cargo_fastqrecord_toolkit-* d -c CONTAINER -n DATASET -t THREADS \
    -o OUT.fastq
```

where `cargo_fastqrecord_toolkit-*` is any FASTQ compressor/decompressor corresponding to one of the implemented *CARGO* methods.
